# An epistatic effect of KRT25 on SP6 is involved in curly coat in horses

**DOI:** 10.1038/s41598-018-24865-3

**Published:** 2018-04-23

**Authors:** Annika Thomer, Maren Gottschalk, Anna Christmann, Fanny Naccache, Klaus Jung, Marion Hewicker-Trautwein, Ottmar Distl, Julia Metzger

**Affiliations:** 1 Institute for Animal Breeding and Genetics, University of Veterinary Medicine Hannover Foundation, Hannover, 30559 Germany; 2 Department of Pathology, University of Veterinary Medicine Hannover Foundation, Hannover, 30559 Germany

## Abstract

Curly coat represents an extraordinary type of coat in horses, particularly seen in American Bashkir Curly Horses and Missouri Foxtrotters. In some horses with curly coat, a hypotrichosis of variable extent was observed, making the phenotype appear more complex. In our study, we aimed at investigating the genetic background of curly coat with and without hypotrichosis using high density bead chip genotype and next generation sequencing data. Genome-wide association analysis detected significant signals (p = 1.412 × 10^−05^–1.102 × 10^−08^) on horse chromosome 11 at 22–35 Mb. In this significantly associated region, six missense variants were filtered out from whole-genome sequencing data of three curly coated horses of which two variants within *KRT25* and *SP6* could explain all hair phenotypes. Horses heterozygous or homozygous only for *KRT25* variant showed curly coat and hypotrichosis, whereas horses with *SP6* variant only, exhibited curly coat without hypotrichosis. Horses with mutant alleles in both variants developed curly hair and hypotrichosis. Thus, mutant *KRT25* allele is masking *SP6* allele effect, indicative for epistasis of *KRT25* variant over SP6 variant. In summary, genetic variants in two different genes, *KRT25* and *SP6*, are responsible for curly hair. All horses with *KRT25* variant are additionally hypotrichotic due to the *KRT25* epistatic effect on *SP6*.

## Introduction

Horse coats and their specific types and colors represent one of the most important characteristics of different breeds and populations after thousands of years of selective breeding in the course of domestication^1,2^. Thus, the composition and thickness of hair does not only play an essential role in the protection from heat or other physical or chemical influences but is also a distinctive feature to define modern horse breeds^3^. In particular, curly coat represents an outstanding feature that does not only occur in various types of horse breeds but is also unique due to its hypoallergenic potential resulting in milder or even no allergic symptoms in several horse allergic patients^4–6^. Curly coated horses have been found to develop a varying degree of curliness based on seasonal influences and in some cases to shed mane and tail in the summer or even develop a persistent hypotrichosis^7^. Histologic investigations of hypotrichotic curly horses revealed that this phenotype represents a form of follicular dysplasia^7^.

The mode of inheritance for curly coat is controversially discussed. Segregation ratios in matings of curly with straight coated Percheron as well as among curly coated Lokai horses suggested an autosomal recessive inheritance^5,6^. Analyses of breeding records of the American Bashkir Curly registry indicated an autosomal dominant mode of inheritance for curly coat as curly coated stallions sired curly and straight coated foals with curly coated mares^8,9^. These findings led to the suggestion that there might be two genetic types involved in the development of curly coat whose occurrence is dependent on breed or regional distributions of horses^10^.

The genetic cause for dominant curly coat was suggested to be a mutation derived from feral horses of North America^10^. Cross breedings with Quarter Horses (QH), Appaloosa and Paint Horses strongly influenced the development of a horse breed specifically selected for this curly coat trait, the so called American Bashkir Curly Horse (ABCH)^10^.

Nevertheless, curly coat has not only been found in horses but also in other species like cats^11,12^, cattle^13^, dogs^14^, rats^15^, mice^16^, rabbit^17^, pigs^18^ and humans^19^. In cats, several Rex breeds developed curly coat hair and vibrissae as a breed defining trait^11,12,20^. In Devon and Cornish Rex, the mode of inheritance was suggested to be autosomal recessive whereas an autosomal dominant locus was demonstrated for the tightly curled coat in Selkirk Rex cats^11,12,20^. Similar rexoid hair types were found in rat and mouse mutants in which a dominantly inherited mutation was proposed to result in curly hair and even in hair loss in homozygous rat mutants^15,21^. The rexoid phenotype in mice was shown to be indistinguishable from the Caracul curly coat type^22^, which could also be observed in Swedish cattle^23^. Other curly coats were found in Fleckvieh and Montbeliarde cattle and were also suggested to be dominantly inherited^13^. In humans, various curly hair types have been described either as specific morphologic types in populations or related with disorders^24–26^. There is evidence for a woolly hair type in men characterized by coarse, lusterless and tightly curled hair inherited as an autosomal dominant or recessive trait^25,26^. Affected patients showed different degrees of hypotrichosis similar to some curly coated horses. A so-called “scanty tail” was described in curly horses with remaining hair fibers at the root whereas a “string tail” with only few hair fibers at the tip of the tail was found in more severely affected horses^7,25^.

And even though curls in the coat of horses are popular when they occur not only in ABCHs but also in other horse breeds^5,6^, the genetic cause for curls in horses and for hypotrichosis in some individuals has not been discovered so far. In this study, we performed genotyping on a high density bead chip and next generation sequencing to identify causative variants for the development of curly coat and in addition, to disentangle the genetic mechanism for hypotrichosis in curly coated horses.

## Results

### Genome- and chromosome-wide association analysis

**Figure 1:**
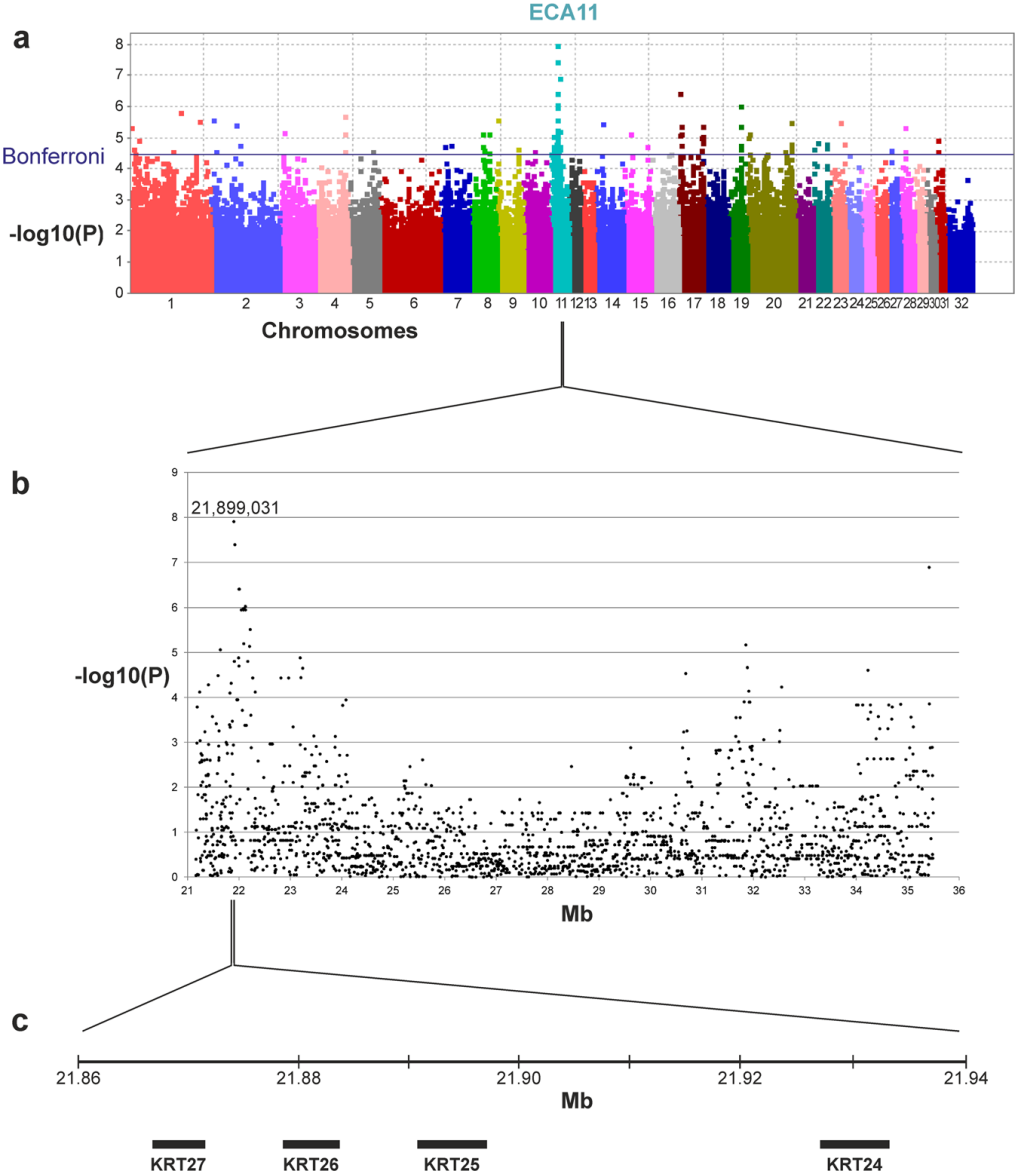
Genome-wide association analysis for curly versus straight coat. (**a**) Manhattan plot of −log10 p-values shows the highest and significant peak region on equine chromosome (ECA) 11. (**b**) Depiction of −log10 P-values in the region of the highest association at 21.9–35.4 Mb including the peak at 21,899,031 bp (P = 7.958). (**c**) Magnification of the region spanning the SNP with the highest p-value at 21,899,031 bp. From a total number of 202 genes found in the whole region of association, *KRT25* could be identified closest to the most significant SNP.

In our study, we phenotyped 216 horses and classified them into horses with curly coat accompanied with complete hypotrichosis, horses with curly coat accompanied with incomplete hypotrichosis, horses with curly coat but without hypotrichosis and straight coated horses without hypotrichosis. All horses with curly coat exhibited a curly tail and mane hair, too. The shape and tightness of curls in the coat was analogous to the shape and tightness of curls in mane and tail. The individual hair fibers had a more rough appearance in curly coated and hypotrichotic horses when compared with just curly coated horses. From these samples we chose 28 curly and 20 straight coated horses including ABCHs and Missouri Foxtrotters for genotyping on the Axiom Equine Genotyping Array 670 K (Affymetrix; Supplementary Table S1). Genome-wide association analysis for curly coat showed a highly significant peak on equine chromosome (ECA) 11 at 21,899,031 to 35,414,844 bp (Fig. 1a). In this peak region, 26 SNPs reached the significance threshold after correction for multiple testing using a Bonferroni correction. The highest association was found at 21,899,031 bp (p = 1.102 × 10^−8^) for AX-104299273 (Fig. 1b).

Genotyping of the seven highest associated SNPs (Supplementary Table S2) in additional 139 horses and subsequent imputation onto all Axiom genotypes on ECA11 in these horses (Supplementary Tables S3–S4) confirmed the associated region of 13.52 Mb in size. This genomic region harbored 202 genes including the two *keratin (KRT)* genes *KRT24* and *KRT25* and further 15 keratin genes located proximal of this region of association (Fig. 1c).

A genome-wide association analysis using horses with hypotrichosis as cases and horses without hypotrichosis as controls also revealed a genome-wide significant peak on ECA11 in the same region at 21,579,177–24,075,050 bp with the most significant SNP at 22,122,892 bp (p = 3.96 × 10^−6^, Supplementary Table S5).

### Whole-genome sequencing

Whole-genome sequence data of one curly coated ABCH with complete hypotrichosis, one curly coated ABCH with incomplete hypotrichosis and one Missouri Foxtrotter without hypotrichosis as well as 27 straight coated controls revealed six variants located in associated region and the proximal keratin cluster (21,162,881–35,414,844 bp) with high or moderate effects exclusively found in one, two or all three curly coated horses (Supplementary Table S6). Pedigree analyses revealed curly coat-associated haplotypes in investigated families (Supplementary Figs S1–S2).

**Figure 2:**
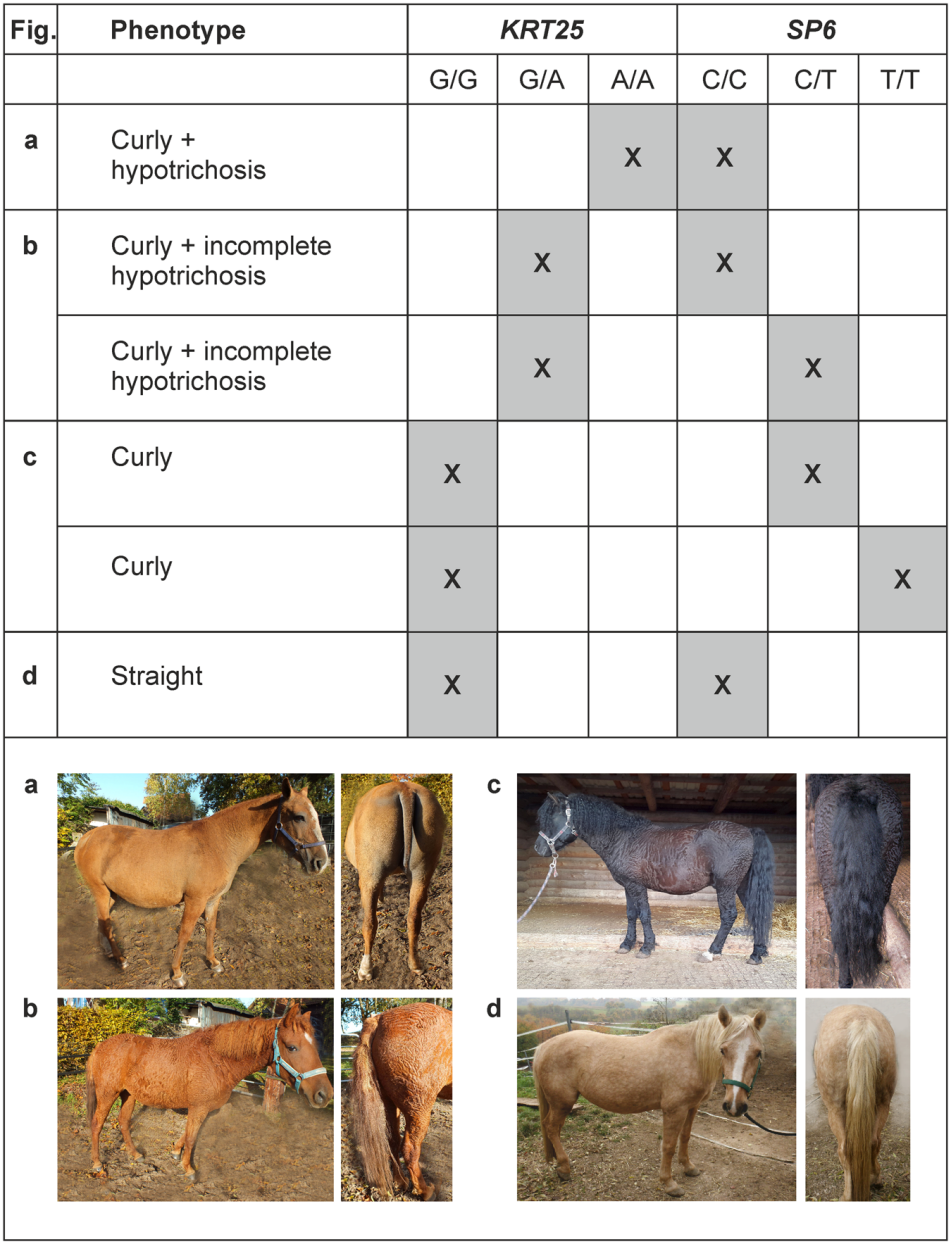
Joint distribution of the genotypes of *KRT25* and *SP6* variants demonstrating the epistatic effect of *KRT25*. The *KRT25* variant NC_009154.2:g.21891160G>A is phenotypically fully expressed despite the presence of *SP6* variant NC_009154.2:g.24022045C>T. A curly coated horse with complete hypotrichosis is shown to harbor a homozygous mutant *KRT25* genotype (**a**). Curly horses with incomplete hypotrichosis show a mutant allele only in *KRT25* variant or both in *KRT25* and *SP6* variant (**b**). Curly horses with no hypotrichosis have either a heterozygous or homozygous mutant genotype for *SP6* variant (**c**). Straight coated horses have a homozygous wild type genotype in both loci (**d**). Grey coloring and crosses mark the genotypic distribution identified in this study and its correlation with the phenotypes.

Validation of all six variants in 148 curly and 68 straight coated horses revealed three genetic variants located within *KRT25* (NC_009154.2:g.21891160G>A, ss2137510528), *transcription factor Sp6* (*SP6*; NC_009154.2:g.24022045C>T, ss3021042887) and *keratin associated protein 16* (*KRTAP16*, NC_009154.2:g.21414219G>A, ss2137510527) segregating with the curly phenotype (Supplementary Table S7). After genotyping of these three variants in a larger validation sample of 17 different equine populations, we found the *KRTAP16* variant segregating in straight coated horses and therefore excluded this variant as causative for curly coat. The joint genotypic distribution of *KRT25* and *SP6* variants explained all curly phenotypes (Table 1). Horses with curls were heterozygous or homozygous in mutant alleles either in *KRT25* or in *SP6* variant or in both variants indicating a complete dominant allele effect for both curly mutations (Fig. 2). In addition, individuals with mutant *KRT25* variant exhibited a variable degree of hypotrichosis (Supplementary Figs S3–S8). Horses with a homozygous mutant genotype for *KRT25* variant showed not only few and sparse curly hair of rough appearance but also an extreme shedding (hypotrichosis) whereas horses with a heterozygous mutant *KRT25* genotype revealed an incomplete hypotrichosis and curly hair of rough appearance regardless of the *SP6* mutant genotype. In contrast, horses exclusively heterozygous or homozygous in *SP6* variant showed curly coat without hypotrichosis and were phenotypically indistinguishable. Most of these horses were derived from Missouri Foxtrotter breed or revealed Missouri Foxtrotter ancestors. Thus, *KRT25* variant was epistatic to *SP6* variant masking the effect of this variant by promoting rough hair and hypotrichosis.

**Table 1:** Genotypic distribution of *KRT25, SP6* and *KRTAP16* missense variants in all investigated equine populations.

Breed/population	Coat	Hypotrichosis	n	NC_009154.2:g.218911 60G>A (*KRT25*)	NC_009154.2:g. 24022045C>T (*SP6*)	NC_009154.2:g.2141421 9G>A *(KRTAP16)*
American Bashkir Curly Horse	Curly	Complete	22	**A/A**	C/C	G/G
American Bashkir Curly Horse	Curly	Incomplete	85	G/**A**	C/C	G/G
American Bashkir Curly Horse	Curly	Incomplete	1	G/**A**	C/C	G/**A**
American Bashkir Curly Horse	Curly	Not at all	3	G/G	**T/T**	**A/A**
American Bashkir Curly Horse	Curly	Not at all	15	G/G	C/**T**	G/**A**
American Bashkir Curly Horse	Curly	Not at all	1	G/G	C/**T**	**A/A**
American Bashkir Curly Horse	Curly	Incomplete	6	G/**A**	C/**T**	G/**A**
American Bashkir Curly Horse	Straight	Not at all	23	G/G	C/C	G/G
American Bashkir Curly Horse	Straight	Not at all	1	G/G	C/C	G/A
Miniature American Bashkir Curly Horse	Curly	Complete	1	**A/A**	C/C	G/G
Miniature American Bashkir Curly Horse	Curly	Incomplete	2	G/**A**	C/C	G/G
Miniature American Bashkir Curly Horse	Curly	Incomplete	1	G/**A**	C/**T**	G**/A**
Miniature American Bashkir Curly Horse	Curly	Not at all	1	G/G	C/**T**	G/**A**
American Bashkir Curly Horse - Quarter Horse	Curly	Incomplete	1	G/**A**	C/C	G/G
Quarter Horse	Straight	Not at all	39	G/G	C/C	G/G
American Bashkir Curly Horse - Paint Horse	Straight	Not at all	1	G/G	C/C	G/G
Kentucky Mountain Saddle Horse	Curly	Incomplete	1	G/**A**	C/C	G/G
Missouri Foxtrotter	Curly	Not at all	5	G/G	C/**T**	G/**A**
Missouri Foxtrotter	Straight	Not at all	3	G/G	C/C	G/G
Danish Warmblood	Curly	Not at all	1	G/G	C/**T**	G/**A**
Oldenburger	Curly	Incomplete	1	G/**A**	C/C	G/G
Oldenburger	Straight	Not at all	1	G/G	C/C	G/G
Holsteiner	Curly	Incomplete	1	G/**A**	C/C	G/G
Holsteiner	Straight	Not at all	1	G/G	C/C	G/G
Hanoverian	Straight	Not at all	13	G/G	C/C	G/G
Duelmen horse	Straight	Not at all	8	G/G	C/C	G/G
Black Forest Coldblood horse	Straight	Not at all	7	G/G	C/C	G/G
Norwegian	Straight	Not at all	1	G/G	C/C	G/G
Lewitzer	Straight	Not at all	8	G/G	C/C	G/G
Friesian	Straight	Not at all	8	G/G	C/C	G/G
Miniature Donkey	Straight	Not at all	4	G/G	C/C	G/G
Sorraia	Straight	Not at all	2	G/G	C/C	G/G
Standardbred	Straight	Not at all	1	G/G	C/C	G/G
Przewalski horse	Straight	Not at all	2	G/G	C/C	G/G
Rhenish German Coldblood	Straight	Not at all	7	G/G	C/C	G/G
Arabian Thoroughbred	Straight	Not at all	319	G/G	C/C	G/G
Arabian Thoroughbred	Straight	Not at all	26	G/G	C/C	G/**A**
Arabian Thoroughbred	Straight	Not at all	2	G/G	C/C	**A/A**
Anglo-Arabian	Straight	Not at all	1	G/G	C/C	G/G
Austrian Coldblood	Straight	Not at all	8	G/G	C/C	G/G
Swedish Warmblood	Straight	Not at all	1	G/G	C/C	G/G
Trakehner-Barb horse	Straight	Not at all	1	G/G	C/C	G/G

Coat type, clinical hypotrichosis and genotypes are shown. Mutant alleles are printed in bold.

Both the substitution of arginine to histidine in KRT25 protein and the substitution of glycine to serine in SP6 protein were predicted to be possibly damaging (KRT25: 1.00, Sp6: 0.84) by PolyPhen-2^27^ as well as deleterious (KRT25: 0.01) and tolerated/neutral (SP6: 0.15/0.83) by SIFT^28^. In addition, SP6 variant was predicted to be located in a low complexity region (position 361–373, Ensembl release 91).

**Figure 3:**
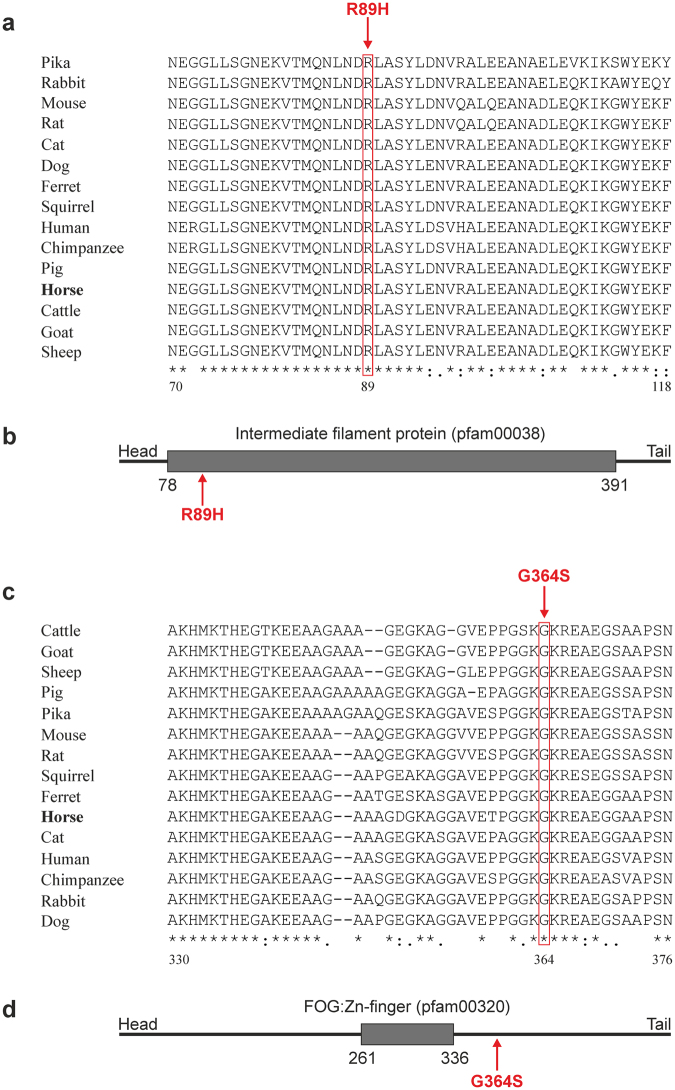
Protein sequence alignment and domains. (**a**) Alignment of KRT25 protein sequence using Clustal Omega. Positions with a fully conserved residue (asterisks) or a conservation of strongly similar (colons) as well as weakly similar properties (periods) between groups are displayed. The missense variant R89H occurs in a highly conserved region. (**b**) Predicted protein domain of KRT25. The variant R89H is located in the intermediate filament protein domain. (**c**) Alignment of SP6 protein sequence using Clustal Omega. The missense variant G364S is also located in a conserved region. (**d**) Predicted protein domain of SP6. The variant G364S is can be found distal of the FOG:Zn-finger domain.

This was reassured using comparative species alignments with Clustal Omega^29^ showing *KRT25* variant to be located in a highly conserved region which is located within the predicted intermediate filament protein domain (Fig. 3a,b). Alignments of *SP6* revealed the *SP6* variant in a conserved section of the protein distal of the Zn-finger domain friend of GATA (FOG) family (Fig. 3c,d).

### RNA-Seq and expression analysis

To further address functional aspects regarding curly coat development and hypotrichosis, RNA sequencing was performed in parallel to whole-genome sequencing analysis in nine curly coated ABCHs, three straight coated ABCHs and three straight coated QHs (Supplementary Table S8). It resulted in an average number of 77 million mapped reads per sample, an average number of five billion bases and an average quality (Phred quality score) of 34. Differential gene expression analysis for curly versus straight hair revealed a high number of upregulated genes implied by negative log fold changes (logFC) in curly ABCHs and downregulated genes implied by positive logFC in curly ABCHs (Supplementary Table S9). Filtering for significant FDR adjusted p-values (padj < 0.05) showed in 156 differentially expressed genes (DEGs). In total, 42 of these significant DEGs were located on ECA11 and were all upregulated in curly coated horses.

Further investigation of the two genes *KRT25* and *SP6* revealed no differential expression of their transcripts. Nevertheless, we identified six keratin genes and further 14 hair development related genes with significant p-values in close proximity of *KRT25* and *SP6*. To explore the potential interaction of these genes due to their co-localization, validation of their expression levels was performed in additional 38 horses (Supplementary Table S10). A generalized linear model (GLM) analysis used to find out whether there was a relation of *KRT25* or *SP6* genotypes with the expression levels of the investigated 20 genes revealed significant p-values for *keratin 17 (KRT17, p(FDR)* = *0.04)* and *SRY-box 9 (SOX9, p(FDR)* = *0.0005)*. Gene network analysis predicted a co-expression of *SOX9* with *KRT25* and *KRT17* and further co-expression of *SOX9, KRT25, KRT17* and *SP6* with various further keratin genes (Supplementary Fig. S9).

In addition to the analysis of genes in close proximity of the candidate variants on ECA11, we investigated the total set of 156 DEGs for potential interactors with *KRT25* or *SP6* (Supplementary Table S11). Only one of the 20 predicted *KRT25*-interactors, the keratin gene *KRT1*, was found to be significantly differentially expressed in curly versus straight horses. Further group comparison of horses with mutant *KRT25* versus *KRT25* wild genotypes revealed an even higher significance for the differential expression of *KRT1* and in addition a significant p-value for *KRT79*. Comparisons of horses with mutant *SP6* versus *SP6* wild genotypes revealed none of the 10 *SP6*-interactors or 20 *KRT25*-interactors to be significantly differentially expressed.

### Morphologic analysis

**Figure 4:**
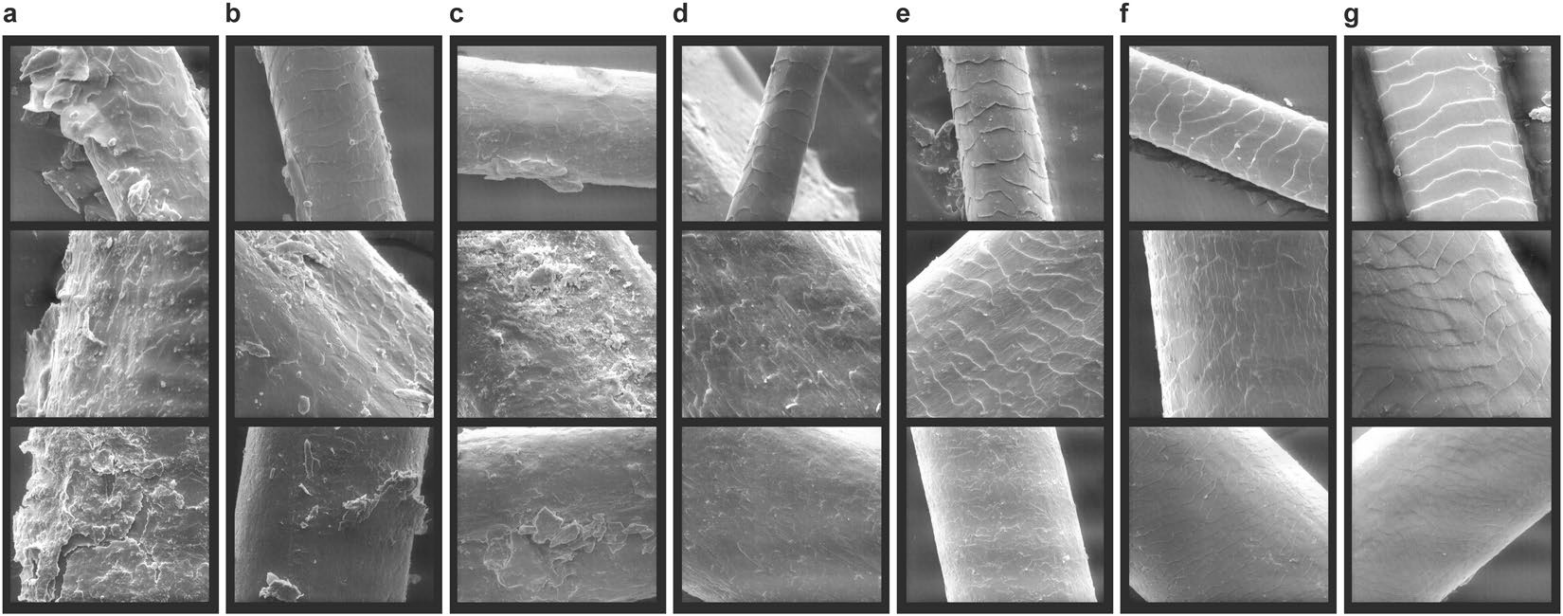
Morphologic characterization of curly genotypes. Scanning electron microscopy (SEM) imaging of tail (500x), mane (1000x) and coat hair (1000x) (descending per column). Curly hair fibers from horses homozygous mutant for *KRT25* variant display an extremely irregular desquamation with detached and stacked scales (**a**). Hair samples from horses with a heterozygous *KRT25* genotype (**b**) or a heterozygous *KRT25* and *SP6* genotype (**c**) also show an irregular desquamation but a less pronounced detachment of scales. In contrast, hair fibers of horses with two mutant *SP6* alleles (**d**) or one mutant *SP6* allele (**e**) appear to have a regular desquamation and only slightly raised scales. A sharply defined and regular desquamation is shown in straight hair of ABCHs (**f**) as well as of QHs (**g**).

High-resolution scanning of hair surfaces of coat, mane and tail hair from horses of all six different detected *KRT25* and *SP6* genotype combinations showed that horses only mutated for *KRT25* could be clearly distinguished from horse only mutated for *SP6*. However, horses heterozygous for both variants were indistinguishable from horses only mutated for *KRT25* (Fig. 4). The hair surface of *KRT25* mutants as well as *KRT25* and *SP6* mutants was rough, irregularly desquamated and scaly. Homozygous *KRT25* mutant horses (A/A) particularly developed an extremely irregular desquamation as well as scales partially detached from the shaft. Some scales were raised in these hair samples resulting in thickened areas due to a stacking of scales. In contrast, hair fibers of curly coated horses mutant for *SP6* variant (C/T or T/T) but wild type in *KRT25* revealed a regular desquamation with only slightly raised scales. In straight coated horses and straight coated QHs the desquamation was regular as well, and the surface of hair fibers was continuously smooth.

The outer appearance of curly hair investigated by scanning electron microscopy (SEM) revealed depressions and rotations in horses mutant for *KRT25*, *SP6* or both. In contrast, straight hair had a fully cylindrical shape without depressions or rotations (Supplementary Fig. S10).

Cross sections of middle parts of guard hair fibers showed a significantly different appearance in curly hair in comparison to straight hair. Curly hair samples of horses mutant for *KRT25*, *SP6* or both genes showed a heterogeneous but not circular shaped shaft and a restricted medulla region (Supplementary Fig. S11). No apparent differences were identified in-between horses with homozygous mutant *KRT25* or *SP6* (A/A or T/T) genotypes and horses with heterozygous genotypes. In contrast, sections of hair shafts from straight hair had the shape of a symmetric circle and revealed a centered medulla region both in straight ABCHs and QHs. Longitudinal sections supported these findings. Mid regions of curly hair shafts showed only medullar remnants. The depicted structures mostly complied with pure cortex tissue. In straight hair, the medulla was clearly defined showing a clean and sharp transition to the cortex.

## Discussion

Our study clearly demonstrated the independent effects of *KRT25* and *SP6* on curly coat development in horses. Hair fibers from both mutant *SP6* or mutant *KRT25* horses showed typical curly hair characteristics like a polymorphic shape, restricted medulla and rotated shafts with depressions. These findings, which were also identified in human, rat, mice and cattle curl types, were postulated to be essential properties of curly hair^15,23,30–32^. They confirmed a complete dominance for curliness of hair fibers for both variants. In addition, an incomplete or complete hypotrichosis was clinically obvious in horses with mutant *KRT25* as well as in horses with mutant *KRT25* and mutant *SP6*. In these horses, the rough texture of the coat appeared due to an irregular desquamation and frayed scales especially pronounced in horses with complete hypotrichosis probably supporting broken hair fibers and hair loss along with dysplastic hair follicles identified in skin biopsies^7^. We conclude that *KRT25* variant has pleiotropic effects on hair fibers leading to curly hair, a modified hair fiber structure and hypotrichosis. For this reason, we propose that mutant *KRT25* is epistatic to mutant *SP6*, meaning the effect of *SP6* is overlaid due to the pleiotropy of *KRT25* variant on the structure of hair fibers.

Previous reports on curly horse pedigrees already suggested more than one genetic mechanism as the cause for curly hair^9^. An evaluation independent from this study identified the *KRT25* variant in French and North American horses associated with curly hair^33^. In this work, which has been published during the review process of this manuscript, *KRT25*-associated phenotypes were assessed by their outer appearance and assumed to be curly coated only. However, the results of our study show that hypotrichosis and rough hair surface are actually important properties that do not only differentiate the horses from curly hair types not carrying *KRT25* variant but are also essential for the distinction of heterozygous or homozygous mutant genotypes.

Furthermore, it was postulated that there might be a second dominant locus for curly hair in particular in curly horses crossed with Missouri Foxtrotters potentially located in an incorrectly annotated region of the horse reference genome^33^. However, our analysis revealed that *SP6* was located in an annotated region but was more difficult to detect due to the breeders unintentional mixing of horses harboring *KRT25* variant with horses harboring *SP6* variant. Due to the identification of *SP6* variant, we could explain phenotypes of so far discordant horses^33^ without a mutant *KRT25* allele. In addition, we observed a particularly high frequency of *SP6* variant in Missouri Foxtrotters and a mixture of both variants in intermixes of Missouri Foxtrotters and ABCHs whereas *KRT25* variant was more frequent in ABCHs. In all samples used for this study, the number of horses harboring *SP6* variant in comparison to those harboring *KRT25* variant was low. We assume that targeted selection for purebred ABCHs might have led to a wider distribution of *KRT25* variant among curly coated horses. This also supports the results from genome-wide association analysis showing the highest peak of association close to *KRT25* probably due to the larger number of individuals genotyped on the bead chip harboring mutant *KRT25*, in comparison to those harboring mutant *SP6* or both mutant alleles. Further genome-wide association analysis for horses with hypotrichosis compared to horses without hypotrichosis confirmed the region harboring *KRT25*, presumably as the development of hypotrichosis and *KRT25* alleles are in complete concordance.

Both identified missense variants in *KRT25* and *SP6* were predicted to provide a modified protein but did not affect gene expression of *KRT25* or of *SP6*. Nevertheless, a significant increase of *KRT17* expression was observed in curly horses. We propose that this expression might probably be modified due to the increased need of regenerative processes in particular in horses with hair fibers with a worn surface and detached scales. *KRT17* is known to be notably expressed in regenerated hair follicles and is important for hair follicle neogenesis^34^. We assume that the reduced rigidity of hair fibers we identified in morphologic analyses might probably have triggered secondary self-repairing processes performed by *KRT17* and maybe also by *KRT1*, *KRT79* and *SOX9*, due to their involvement in epidermal and hair regeneration processes^35–37^.

Thus, we assume that the development of curly hair is not a result of differential expression but of protein modifying effects as is was postulated in various other curly coated animals^13,14,38^. We identified a high conservation in the regions of mutant *KRT25* and *SP6* alleles suggesting a probably damaging effect on SP6 and KRT25 protein. Effect prediction tools confirmed this assumption even though SP6 was proposed to be possibly damaging or tolerated by different databases. Nevertheless, we identified this variant in a low-complexity region, which was shown to be typically difficult to analyze by conventional sequence analysis procedures^39^. It was postulated that low-complexity regions have a larger number of binding partners in protein interaction networks, which is presumably essential for the transcription factor SP6^39^.

SP6 was shown to play an important role as a highly cell- and tissue-specific transcription factor primary expressed in hair follicles, teeth and limbs^40^. Mice lacking SP6 developed short and curly whiskers whereas the dorsal skin revealed only short hair tips but no development of a dense fur^41^. Thus, it was proposed that SP6 plays an essential role in the proliferation of cells in the skin^40^. We assume that mutant SP6 in curly coated horses might provoke an asymmetry in the proliferative compartment of hair follicles and thereby result in a curved shape of the hair as it was observed in human curly hair^19,30^.

Furthermore, we suppose that mutant *KRT25* might develop curly hair due to a combined effect on hair proliferation in the hair bulb as it was found in human curly hair^30^ and a disorganization of the macrofibril structure. KRT25 was shown to be a member of the type I hair keratins^42^. These keratins were found to be important for the formation of keratin intermediate filaments (microfibrils) as components of macrofibrils essential for the assembly and maintenance of hair structure^42^. A protein modifying variant in human *KRT25* was suggested to cause a disarrangement of the macrofibril structure^43^. Due to its expression in the inner root sheath of the hair follicle and the hair shaft medulla^44^, an altered KRT25 protein in horses is likely to affect all over the formation of macrofibril types resulting in a larger number of orthocortical cells with a typical twisted appearance similar to curly hair fibers in human, instead of homogenous mesocortical cells in straight hair^30,45,46^.

In addition, as it was shown in tightly curled and sparse human woolly hair, the curly phenotype can be related to the development of hypotrichosis and a modified structure of the hair fibers^43^. The hair properties we identified in morphologic analysis can be assumed to be the reason for the coarse and lusterless appearance in these horses just as it was found in human woolly hair caused by a missense variant in *KRT25*^25,43^. Similar observations were made in *KRT25* mouse mutants with ragged and fragile vibrissae^32^ as well as curly coated rexoid mutant rats with an irregularly arranged outer appearance of the hair cuticles all over the body^15^.

In conclusion, we identified two missense variants in *KRT25* and *SP6* acting independently on the development of a curly coat. Moreover, we demonstrated an epistatic effect of *KRT25* variant on SP6 variant due to its pleiotropy on hair structure and hair loss.

## Methods

### Sample Collection and Phenotyping

The study included pedigree data, EDTA-blood and hair samples from 216 horses. All animal work has been operated in compliance with the national and international guidelines for animal welfare. EDTA-blood and hair sampling received the permission of the Lower Saxony state veterinary office Landesamt für Verbraucherschutz und Lebensmittelsicherheit, Oldenburg, Germany (registration number 33.19-42502-05-15A581).

All horses were phenotyped by careful examination of coat, mane, tail, fetlock hair, ear hair and eyelashes. For a reliable identification of phenotypes, all examinations were performed in the winter season when the curly coat was fully expressed. Findings were documented in a questionnaire including the body coat type classified as straight, wavy, medium curl or tight curl. All three types wavy, medium and tight curl were classified as curly coat. Mane and tail were categorized as curly or straight. Furthermore, it was documented whether the horses showed an incomplete or complete hypotrichosis. Hypotrichosis, by definition a loss or reduction of hair, was assigned as complete if the horse showed a total loss of tail and mane hair at the day of examination or at a later time point documented by photos. Horses with incomplete hypotrichosis did not loose all mane and tail hair but showed shedding and broken hair especially at the lateral upper tail and the shock of hair on the head. 148 curly coated individuals composed of 133 American Bashkir Curly Horses, five Miniature ABCHs, one Kentucky Mountain Saddle Horse, five Foxtrotters, one Oldenburger, one Danish Warmblood horses, one Holsteiner and one ABCH QH crossbreed. Samples of 68 straight coated horses comprised 24 ABCHs, 39 QH, three Missouri Foxtrotters, one Oldenburger and one ABCH Paint horse crossbreed. Hair samples were taken from the horses’ tail and stored in RNALater reagent (Qiagen, Hilden, Germany) immediately after sampling.

### Genome- and chromosome-wide association study

For genotyping we isolated genomic DNA from 48 EDTA blood samples with a standard ethanol fraction^47^ and adjusted it to 50 ng/µl. The samples composed of 28 curly coated horses comprising 8 horses with complete hypotrichosis, 15 horses with incomplete hypotrichosis and 5 horses with no hypotrichosis as well as 20 straight coated horses without hypotrichosis were genotyped on the Axiom Equine Genotyping Array 670 K (Affymetrix, Santa Clara, CA, United States) for 670,796 SNPs using standard procedures as recommended by the manufacturer. A minor allele frequency (MAF) of >0.05 and a genotyping rate of 98% served as quality criteria. Genome-wide association analysis was done for curly coated horses as cases versus straight coated horses as controls. In addition, we performed a GWA analysis for horses with hypotrichosis as cases and horses without signs of hypotrichosis as controls.

A max (T) permutation test (mperm 10,000) was done using PLINK, V 1.07 (http://pngu.mgh.harvard.edu/~purcell/plink/). The eigenstrat method was used to test for model robustness using two principal components as covariates (lambda = 1.15587). All −log10 p-values were Bonferroni-corrected using the MULTIPLE TEST procedure of SAS V 9.4 (Statistical Analysis System, Cary, NC, 2017).

Seven SNPs in the peak region of association on ECA11 were further validated in 187 horses, composed of 48 horses already genotyped on the 670 K Axiom Equine Genotyping Array (Affymetrix) and additional 139 horses using competitive allele specific PCR (KASP) genotyping assays (LGC Genomics, Teddington, Middlesex, UK; Supplementary Table S12). These 139 horses could be divided into 88 curly coated ABCHs, one curly coated Miniature ABCH, two curly coated Missouri Foxtrotter, one curly coated Oldenburger, one curly coated Kentucky Saddle Mountain Horse, one curly coated Danish Warmblood, 17 straight coated ABCHs, 25 straight coated QHs, one straight coated Missouri Foxtrotter, one straight coated Oldenburger and one straight coated ABCH Paint Horse crossbreed. KASP genotyping reactions were performed using 5 μl KASP Master Mix 2x (LGC Genomics), 0.14 μl KASP Assay mix (two allele-specific primers, one common primer designed by LGC) and 5 μl template DNA with a concentration of 7–17 ng/µl. After the KASP standard thermal cycling touchdown protocol was run on a thermocycler TProfessional 96 (Biometra, Göttingen, Germany) using an annealing temperature of 61 °C and −0.5 °C decrease in each cycle, allelic discrimination was done on ABI7300 sequence detection system (Applied Biosystems, Waltham, Massachusetts, USA).

In the attempt to narrow down the region of genome-wide association, we used the genotypes of the seven variants validated in 139 additional horses to get more in-depth information of markers in and around this location. We imputed the genotyping results of these seven SNPs onto all Axiom genotypes on ECA11 in 187 individuals for 12,681 SNPs using BEAGLE (V 4.1)^48^.

A chromosome-wide association analysis was run again on basis of these imputed data using PLINK, (V 1.07 (http://pngu.mgh.harvard.edu/~purcell/plink/).

### Whole Genome Sequencing

Whole genome sequencing was performed on Illumina NextSeq 500 (Illumina, San Diego, California, USA) in three curly coated horses including one curly coated ABCH with complete hypotrichosis, one curly coated ABCH with incomplete hypotrichosis and one Missouri Foxtrotter without hypotrichosis. Libraries were prepared from high quality DNA with NEBNext Ultra II DNA Library Prep Kit for Illumina (NEB, Ipswich, MA, USA) using focused-ultrasonicator (Covaris, Woburn, MA, USA) for fragmentation and magnetic beats (AMPure beats, Agilent Technologies, Santa Clara, CA, USA) for size selection. Sequencing was performed in pair-end mode for 300 cycles. Whole-genome sequencing data were submitted to sequence read archive (SRA, NCBI, SubmissionID: SUB2718263, BioProject ID: PRJNA387659). Reads were trimmed with a quality score threshold of 20 (5′ and 3′-end) and a maximum allowed score of 90 using PRINSEQ (V 0.20.4)^49^.

Mapping and variant calling was done for all three curly coated ABCHs and further 27 straight coated controls derived from SRA including six Przewalski horses (SRX305128, SRX305127, SRX302128, SRX302111, SRX302110, SRS441443), three Shetland ponies (ERX947605, ERX947604, SRX1976860), two Connemara Ponies (SRX850675, SRX850674), one Marwari (SRX535352), one Icelandic horse (SRS439179), one Standardbred (SRS438330), one Norwegian Fjord (SRS438157), one Donkey (SRS431817), one Thoroughbred (SRX396629), one Saxon-Thuringian Heavy Warmblood (SRX1131818), four Hanoverian (SRX1131785, SRX1131705, SRX389477, SRX389480), two Sorraia (SRX1131820, SRX389475), two Arabian (SRS431663, SRX389472) and one Duelmen Horse (SRX384479)) using BWA 0.7.13^50^ and SAMtools 1.3.1^51^, Picard tools 2.3.0 (http://broadinstitute.github.io/picard/) and GATK 3.5 (https://software.broadinstitute.org/gatk/)^52^. A minimum read depth of 2 and quality values 20 were applied for further investigated data. Variants were filtered for SNPs or Indels in the candidate region on ECA11, comprising the keratin cluster proximal of the region of association and the highest significant peak of association (21,162,881-35,414,844 bp) using SAS, V 9.4 (Statistical Analysis System, Cary, NC). We specifically selected those variants with a minimum of one mutant allele found in one, two or all three ABCHs and only wild type genotype in the reference horses (Supplementary Table S13). In a second step, those variants with high or moderate effects according to SNPEff predictions (SNPEff V 4.1 g)^53^ were further investigated for their potential influence on protein function using SIFT^28^ and PolyPhen-2^27^.

### Validation of candidate SNPs

Validation of six missense mutations derived from whole-genome sequencing analysis was done using KASP for the NC_009154.2:g.21891160G>A (*KRT25*) variant and restriction fragment length polymorphisms (RFLP) for the remaining five variants in 148 curly coated and 68 straight coated horses (Supplementary Table S14). Graphic representation of haplotypes was constructed using Merlin^54^ and Haplopainter^55^. For the *SP6* variant a mismatch primer was used according to^56^. The three variants, whose genotypes segregated with the phenotypes, NC_009154.2:g.21891160G>A (*KRT25*), NC_009154.2:g.24022045C>T (*SP6*) and NC_009154.2:g.21414219G>A *(KRTAP16)* were further genotyped in 420 equids of 17 populations including Duelmen horse, Black Forest Coldblood, Rhenish German Coldblood, Norwegian, Lewitzer, Friesian, Miniature Donkeys, Sorraia, Trotter, Przewalski, Arabian Thoroughbred, Anglo-Arabian, Austrian Coldblood, Hanoverian, Holsteiner, Trakehner-Barb and Swedish Warmblood (Table 1). All three SNPs were submitted to dbSNP database (http://www.ncbi.nlm.nih.gov/SNP/) referred to as ss3021042887 (NC_009154.2:g.24022045C>T), ss2137510528 (NC_009154.2:g.21891160G>A) and ss 2137510527 (NC_009154.2:g.21414219G>A). In addition, multiple sequence alignment was performed to investigate sequence conservation in the region of the two candidate variants using Clustal Omega^29^ Protein domains were predicted using NCBI conserved domain search^57^.

### RNA Sequencing and Expression Analysis

For RNA sequencing samples of nine curly coated ABCHs, three straight coated ABCHs and three straight coated QHs were selected. Three of the curly coated horses and three of the straight coated horses have already been genotyped on the bead chip for genome-wide association analysis. Total RNA was isolated from coat, mane and tail hair stored in RNALater reagent (Qiagen). For extraction, we used QIAzol Lysis Reagent (Qiagen). Samples were homogenized using Precellys Lysing Kit (VWR International, Darmstadt, Germany) and further processed using RNeasy Lipid Tissue Kit (Qiagen) and RNase-Free DNase Kit (Qiagen) following the manufacturer’s protocol. As quality parameter, only samples with an RNA integrity Number (RIN) of >7 according to RNA Nano Chip quality control (Agilent Technologies) on a Bioanalyzer (Agilent Technologies) were used for analysis. Due to these quality requirements, RNA derived from tail hair was chosen for library preparation, as RIN values were more consistent and RNA concentration was high in comparison to RNA derived from to thinner rooted mane and coat hair samples. Indexed libraries were prepared using TruSeq RNA Library Prep Kit v2 (Illumina) and sequenced on the Illumina NextSeq 500 (Illumina) in paired-end mode for 150 cycles. Fastq-files were quality controlled using fastqc (V 0.11.5). Mapping to the reference genome EquCab 2.0 was performed using STARaligner followed by splitting and trimming using GATK and counting using RSEM-package^58^. Sorting, duplicate marking and indexing was realized using Picard tools. Variant calling was done with HaplotypeCaller^59^.

Raw read counts were normalized as in^60^ and^61^. DEGs between curly coated and straight coated horses were studied by negative binominal tests as described in^60^. The same analyses were performed for group comparisons of mutant *KRT25* versus wild type and mutant *SP6* versus wild type. In order to reduce the proportion of false positive findings, raw p-values were FDR adjusted by Benjamini and Hochberg^62^. Global test procedures were carried out to identify group effects in subsets of genes^63^ related to specific gene ontology (GO) terms^64^. A global test can identify group effects in subsets of genes, even if no single gene is significant itself. All analyses were performed in the statistical programming environment R (V 3.2.2, www.r-project.org). Normalization and differential testing was performed using the R-package DESeq (V 1.22.1), global testing was done using the R-package RepeatedHighDim (V 2.0.0). GO term annotation was retrieved from the Ensembl data base using the R-package ‘biomaRt’^65^.

### Validation of Expression Data

In total, 38 individuals composed of 14 curly coated ABCHs, two curly coated Missouri Foxtrotters, one curly coated Danish Warmblood, one curly coated Holsteiner, 10 straight coated ABCHs, eight straight coated QHs and two straight coated Missouri Foxtrotter were selected for validation. RNA quantity was adjusted to 500 ng and prepared for complementary DNA (cDNA) synthesis with RT2 First Strand Kit (Qiagen). Assays of 21 genes in duplicates were chosen for RT2 Profiler PCR Array plate (Qiagen) design. *GAPDH*, *B2M* and *ACTB* served as housekeepers whereas Genomic DNA Control (GDC) CtGDC >35, Reverse Transcription Control (RTC) and Positive PCR Control (PPC) CtRTC-CtPPC <5, and CtPPC 20 ± 2 were used as quality parameters for each run. Cycles of 95°/10 minutes, 95°/60 minutes and 60°/1 minute (40×) were run on ABI7300 sequence detection system (Applied Biosystems, Foster City, CA, USA). *B2M* was detected to be the most stable housekeeper. In addition, a TaqMan gene expression assay (Applied Biosystems) was applied for *KRT25* as a control sample and *B2M* as housekeeping gene (Supplementary Table S15). Reactions comprising TaqMan expression master mix, assays and cDNA template were run in duplex mode for 40 cycles on ABI7300. Ct values ≥35 were considered a negative call. Level-x values were calculated using ΔΔCT method^66^ for straight coated horses as controls and curly coated horses as cases.

We performed a generalized linear model (GLM) analysis using Statistical Analysis System (SAS/Genetics, V 9.4, SAS Institute) to check the interrelation of the genotypes of the missense variants within *KRT25* and *SP6* and expression data. Genetic interactions were further investigated using GeneMANIA^67^, BioGRID^68^ and IntAct^69^.

### Morphologic analysis

In total, three to four single hair fibers from coat, mane and tail were randomly selected from bunches of hair plucked from 21 horses. Each genotypic combination of KRT25 and SP6 variant occurring in our study population was represented by three individuals (Supplementary Table S16).

All hair samples were investigated using SEM. Mid sections were put onto a conductive glue pad, sputter-coated with a gold layer (Balzers Union SCD 040, Balzers, Liechtenstein, Germany) and scanned using a digital scanning microscope (Zeiss DSM 950, Zeiss, Oberkochen, Germany). All samples were screened thoroughly in 100X, 500X and 1000X magnification.

In addition, cross and longitudinal sections were prepared. The hair pieces were fixed in glutaraldehyde, transferred to 0.1 M cacodylate buffer (SERVA Electrophoresis, Heidelberg, Germany) and postfixed in 1% osmium tetroxide-buffered solution. After that, the samples were dehydrated in ethanol, pre-infiltrated in epon derivate propylene-oxide and embedded. The enclosed sample blocks were incubated at 35 °C and 45 °C for 24 hours each followed by four days at 65 °C. Next, the blocks were cut into 0.5 µm sections on a rotation microtome (Ultracut E, Reichert-Jung, Unterschleissheim, München, Germany), and stained with toluidine blue (Waldeck, Münster, Germany). Coat hair samples could not be cut after embedding due to their too fragile structure. Longitudinal mid hair sections of guard hair fibers were investigated using a light microscope (Olympus BX51, Olympus, Hamburg, Germany) with an Olympus camera DP72 and Olympus cellSens software in 40X and 100X magnification, whereas cross sections were scanned in 200X magnification. Here again, hair samples were compared with each other at mid sections.

### Data availability

Sequence data were submitted to sequence read archive (SRA ID: SRR5591523, SRR5591591, SRR5591598; BioProject ID: PRJNA387659).

## Electronic supplementary material

Supplementary Information

Supplementary Table S9

Supplementary Table S10
