# Supplementary material for: An epistatic effect of KRT25 on SP6 is involved in curly coat in horses
Source: Sci Rep. 2018 Apr 23;8:6374. doi: 10.1038/s41598-018-24865-3 (PMC5913262; doi:10.1038/s41598-018-24865-3)
Supplement: Supplementary file 1 — Supplementary Information [file 41598_2018_24865_MOESM1_ESM.pdf]

An epistatic effect of KRT25 on SP6 is involved in curly coat in horses

Annika Thomer[1], Maren Gottschalk[1], Anna Christmann[1], Fanny Naccache[1], Klaus Jung[1], Marion Hewicker-Trautwein[2], Ottmar Distl[1*], and Julia Metzger[1]

Supplementary Information:

Supplementary Table S1. Animals used for bead chip analysis.
Supplementary Table S2. Genome-wide associated SNPs on ECA11 for curly versus straight horses.
Supplementary Table S3. Chromosome-wide associated SNPs on ECA11 for curly versus straight horses.
Supplementary Table S4. Genotypic distribution of significantly associated SNPs from genome-wide association analysis after validation in further samples.
Supplementary Table S5. Genome-wide associated SNPs on ECA11 for hypotrichosis.
Supplementary Table S6. Filtering results of whole-genome sequencing data.
Supplementary Table S7. Validation of missense variants from filtering analysis of whole-genome sequencing data.
Supplementary Table S8. Phenotypes and mapping parameters for RNA sequencing.
Supplementary Table S9. RNA-Seq results.
Supplementary Table S10. Validation results of differentially expressed genes derived from RNA-Seq.
Supplementary Table S11. Predicted protein interactions.
Supplementary Table S12. Genotyping of significantly genome-wide associated SNPs.
Supplementary Table S13. Results from filtering analysis of whole genome sequencing data.
Supplementary Table S14. Primer sequences used for genotyping of candidate SNPs on ECA11.
Supplementary Table S15. TaqMan gene expression probes used for validation of expression data.
Supplementary Table S16. Animals used for morphologic evaluation.

Figure legends

Figure S1. Segregation of haplotypes in ABCH family.
Figure S2. Segregation of haplotypes in ABCH and crossbreed family.
Figure S3. Pedigree of an American Bashkir Curly Horse (ABCH) family I.
Figure S4. Pedigree of a Missouri Foxtrotter family.
Figure S5. Pedigree of an American Bashkir Curly Horse (ABCH) family II.
Figure S6. Pedigree of an American Bashkir Curly Horse (ABCH) family III.
Figure S7. Pedigree of a crossbreed family.
Figure S8. Pedigree of an American Bashkir Curly Horse (ABCH) family IV.
Figure S9. Gene interaction network.
Figure S10. Scanning of curly and straight hair surface.
Figure S11. Cross- and longitudinal sections.

Table S1. Animals used for bead chip analysis. The individual horses genotyped on the high-density bead chip, their coat type and observed hypotrichosis status is shown.

Breed/population	Sex	Coat	Hypotrichosis
American Bashkir Curly Horse	Female	Curly	Complete
American Bashkir Curly Horse	Female	Curly	Complete
American Bashkir Curly Horse	Female	Curly	Complete
American Bashkir Curly Horse	Female	Curly	Complete
American Bashkir Curly Horse	Female	Curly	Complete
American Bashkir Curly Horse	Male	Curly	Complete
American Bashkir Curly Horse	Male	Curly	Complete
American Bashkir Curly Horse	Female	Curly	Complete
American Bashkir Curly Horse	Male	Curly	Incomplete
American Bashkir Curly Horse	Female	Curly	Incomplete
American Bashkir Curly Horse	Male	Curly	Incomplete
American Bashkir Curly Horse	Female	Curly	Incomplete
American Bashkir Curly Horse	Male	Curly	Incomplete
American Bashkir Curly Horse	Male	Curly	Incomplete
American Bashkir Curly Horse	Female	Curly	Incomplete
American Bashkir Curly Horse	Female	Curly	Incomplete
American Bashkir Curly Horse	Male	Curly	Incomplete
American Bashkir Curly Horse	Female	Curly	Incomplete
American Bashkir Curly Horse	Female	Curly	Incomplete
American Bashkir Curly Horse	Male	Curly	Incomplete
American Bashkir Curly Horse	Female	Curly	Incomplete
American Bashkir Curly Horse	Female	Curly	Incomplete
Holsteiner	Female	Curly	Incomplete
American Bashkir Curly Horse	Male	Curly	Not at all
American Bashkir Curly Horse	Male	Curly	Not at all
Missouri Foxtrotter	Male	Curly	Not at all
Missouri Foxtrotter	Male	Curly	Not at all
Missouri Foxtrotter	Female	Curly	Not at all
American Bashkir Curly Horse	Female	Straight	Not at all
American Bashkir Curly Horse	Female	Straight	Not at all
American Bashkir Curly Horse	Female	Straight	Not at all
American Bashkir Curly Horse	Female	Straight	Not at all
American Bashkir Curly Horse	Female	Straight	Not at all
American Bashkir Curly Horse	Male	Straight	Not at all
Missouri Foxtrotter	Female	Straight	Not at all
Missouri Foxtrotter	Female	Straight	Not at all
Quarter Horse	Female	Straight	Not at all
Quarter Horse	Female	Straight	Not at all
Quarter Horse	Female	Straight	Not at all
Quarter Horse	Female	Straight	Not at all
Quarter Horse	Female	Straight	Not at all
Quarter Horse	Male	Straight	Not at all
Quarter Horse	Male	Straight	Not at all
Quarter Horse	Male	Straight	Not at all

Quarter Horse	Female	Straight	Not at all
Quarter Horse	Male	Straight	Not at all
Quarter Horse	Female	Straight	Not at all
Quarter Horse	Female	Straight	Not at all

Table S2. Genome-wide associated SNPs on ECA11 for curly versus straight horses. The seven highest significantly associated SNPs on ECA11 derived from genome-wide association analysis for curly versus straight horses, their position, SNP ID, minor allele, minor allele frequency for all individuals, cases and controls and P-values (raw and Bonferroni corrected) in 28 cases and 20 controls are shown.

**Position**	**SNP ID**	**Minor allele**	**MAF**			**$-\log_{10}P_{raw}$**	**$-\log_{10}P_{Bonf}$**
			**all**	**cases**	**controls**	**P_{raw}**	**P_{Bonf}**
21899031	AX-104299273	G	0.438	0.160	0.739	7.958<br>$1.102\ 10^{-08}$	5.890<br>$1.289\ 10^{-06}$
21907825	AX-104617737	A	0.365	0.160	0.587	4.850<br>$1.412\ 10^{-05}$	2.782<br>0.002
21910866	AX-103834321	C	0.365	0.160	0.587	4.850<br>$1.412\ 10^{-05}$	2.782<br>0.002
21922261	AX-104641671	A	0.490	0.220	0.783	7.442<br>$3.612\ 10^{-08}$	5.374<br>$4.226\ 10^{-06}$
21999530	AX-104311931	T	0.469	0.220	0.739	6.451<br>$3.544\ 10^{-07}$	4.382<br>$4.146\ 10^{-05}$
22006773	AX-103796906	A	0.469	0.220	0.739	6.451<br>$3.544\ 10^{-07}$	4.382<br>$4.146\ 10^{-05}$
35414844	AX-104824172	T	0.479	0.220	0.761	6.935<br>$1.162\ 10^{-07}$	4.867<br>$1.360\ 10^{-05}$

Table S3. Chromosome-wide associated SNPs on ECA11 for curly versus straight horses. Chromosome-wide association analysis for curly versus straight horses was performed after imputation of ECA11 in further 137 horses onto all Axiom genotypes. It confirmed a high association for all seven SNPs, which were also highly associated in genome-wide association analysis. Position, SNP ID, minor allele, minor allele frequency for all individuals, cases and controls, and P-values (raw and Bonferroni corrected) are shown.

**Position**	**SNP ID**	**Minor allele**	**MAF**			**$–log_{10}P_{raw}$**	**$–log_{10}P_{Bonf}$**
			**all**	**cases**	**controls**	**P_{raw}**	**P_{raw}**
21899031	AX-104299273	G	0.447	0.167	0.739	13.519<br>$3.030\ 10^{-14}$	9.416<br>$3.840\ 10^{-10}$
21907825	AX-104617737	A	0.365	0.160	0.587	10.423<br>$3.780\ 10^{-11}$	6.320<br>$4.790\ 10^{-07}$
21910866	AX-103834321	C	0.365	0.160	0.587	10.423<br>$3.780\ 10^{-11}$	6.320<br>$4.790\ 10^{-07}$
21922261	AX-104641671	A	0.500	0.229	0.783	18.414<br>$3.850\ 10^{-19}$	14.311<br>$4.880\ 10^{-15}$
21999530	AX-104311931	T	0.469	0.220	0.739	17.578<br>$2.640\ 10^{-18}$	13.475<br>$3.350\ 10^{-14}$
22006773	AX-103796906	A	0.469	0.220	0.739	17.417<br>$3.830\ 10^{-18}$	13.314<br>$4.860\ 10^{-14}$
35414844	AX-104824172	T	0.479	0.220	0.761	14.183<br>$6.570\ 10^{-15}$	10.080<br>$8.330\ 10^{-11}$

Table S4. Genotypic distribution of significantly associated SNPs from genome-wide association analysis after validation in further samples. The distribution is shown for 122 curly coated and 65 straight coated horses. SNP position on ECA11, SNP ID, minor allele frequency and number of animals (N) in cases (%) per locus are presented. The distribution of genotypes is accessed through minor allele (0) and major allele (1) allocation in all cases representing curly coated individuals.

**SNP position**	**SNP ID**	**Minor allele frequency**	**Number of animals (N) in cases (%)**	**Genotype**		
				**0/0**	**0/1**	**1/1**
11-21899031	AX-104299273	0.44	N	35	95	57
			cases (%)	0.0	68.4	86.0
11-21907825	AX-104617737	0.39	N	27	91	69
			cases (%)	0.0	63.7	81.2
11-21910866	AX-103834321	0.39	N	27	91	69
			cases (%)	0.0	63.7	81.2
11-21922261	AX-104641671	0.49	N	43	98	46
			cases (%)	0.0	71.4	95.7
11-21999530	AX-104311931	0.48	N	42	95	50
			cases (%)	0.0	71.6	92.0
11-22006773	AX-103796906	0.47	N	42	93	52
			cases (%)	0.0	72.0	90.4
11-35414844	AX-104824172	0.48	N	45	90	52
			cases (%)	13.3	67.8	90.4

Table S5. Genome-wide associated SNPs on ECA11 for hypotrichosis. The highest significantly associated SNPs on ECA11 derived from genome-wide association analysis for horses with hypotrichosis versus horses without hypotrichosis, their position, SNP ID, minor allele, minor allele frequency for all individuals, cases and controls and P-values (raw and Bonferroni corrected) in 28 cases and 20 controls are shown.

**Position**	**SNP ID**	**Minor allele**	**MAF**			**$–log_{10}P_{raw}$**	**$–log_{10}P_{Bonf}$**
			**all**	**cases**	**controls**	**P_{raw}**	**P_{Bonf}**
22122893	AX-103392490	T	0.385	0.060	0.739	11.071<br>$8.487\ 10^{-12}$	5.403<br>$3.958\ 10^{-06}$
21579177	AX-103918845	T	0.479	0.740	0.196	7.016<br>$9.634\ 10^{-08}$	1.347<br>0.04493
21751182	AX-104423087	T	0.479	0.740	0.196	7.016<br>$9.634\ 10^{-08}$	1.347<br>0.04493
21828222	AX-104243153	G	0.448	0.160	0.761	8.477<br>$3.332\ 10^{-09}$	2.808<br>0.001554
21865941	AX-104659057	T	0.427	0.160	0.717	7.459<br>$3.478\ 10^{-08}$	1.790<br>0.01622
21870036	AX-103043342	G	0.406	0.140	0.696	7.514<br>$3.064\ 10^{-08}$	1.845<br>0.01429
21899031	AX-104299273	G	0.436	0.700	0.152	7.189<br>$6.47\ 10^{-08}$	1.520<br>0.03018
22171821	AX-104272517	T	0.365	0.620	0.087	7.227<br>$5.933\ 10^{-08}$	1.558<br>0.02767
22239354	AX-103741142	G	0.417	0.700	0.109	8.362<br>$4.341\ 10^{-09}$	2.694<br>0.002025
23191174	AX-104221529	C	0.479	0.200	0.783	7.943<br>$1.141\ 10^{-08}$	2.274<br>0.00532
23238823	AX-103795090	A	0.396	0.100	0.717	8.082<br>$6.43\ 10^{-10}$	3.523<br>0.0002999
23453288	AX-105006289	G	0.436	0.146	0.739	8.174<br>$6.696\ 10^{-09}$	2.505<br>0.003123
23502347	AX-103118220	G	0.271	0.021	0.522	7.153	1.484

						7.033 10^{-08}	0.03281
23628626	AX-103862933	G	0.490	0.760	0.196	7.484	1.816
						3.279 10^{-08}	0.01529
24075050	AX-103477609	C	0.417	0.680	0.130	7.313	1.644
						4.863 10^{-08}	0.02268

Table S6. **Filtering results of whole-genome sequencing data.** Six missense mutations derived from filtering analysis for variants on ECA11 in the region of the keratin cluster proximal of the region of association and in the peak region of association (21,162,881-35,414,844 bp) are shown. Only variants with predicted high or moderate effects exclusively found heterozygous (0/1) or homozygous mutant (1/1) in one, two or all three curly coated horses and not detected in any of the straight coated reference horses were selected. Variant effects were further estimated using SIFT and PolyPhen-2.

**Variant**	**Amino acid change**	**Consequence**	**Genotype Horse 1**	**Genotype Horse 2**	**Genotype Horse 3**	**Gene (transcript)**	**SIFT**	**PolyPhen-2**
NC_009154.2:g.21338314G>C	Gly>Ala	missense variant	0/1	0/0	1/1	*ENSECAG00000014468* (ENSECAT00000015098)	tolerated (0.20)	benign (0.008)
NC_009154.2:g.21414219G>A	Val>Ile	missense variant	0/1	0/0	1/1	*KRTAP16*, LOC100066923 (ENSECAT00000010682)	tolerated (1.00)	benign (0.000)
NC_009154.2:g.21891160G>A	Arg>His	missense variant	0/1	1/1	0/0	*KRT25* (ENSECAT00000014491)	deleterious (0.01)	possibly damaging (1.000)
NC_009154.2:g.22186465C>T	Ser>Leu	missense variant	0/1	0/1	0/0	*TOP2A* (ENSECAT00000014604)	deleterious (0.05)	possibly damaging (0.903)
NC_009154.2:g.24022045C>T	Gly>Ser	missense variant	0/1	0/0	1/1	*SP6* (ENSECAT00000013302)	tolerated (0.15)	possibly damaging (0.840)
NC_009154.2:g.26187590C>T	Pro>Leu	missense variant	0/1	0/1	0/0	*EPN3* (ENSECAT00000005184)	deleterious (0.00)	possibly damaging (1.000)

Table S7. Validation of missense variants from filtering analysis of whole-genome sequencing data. The genotypic distribution of investigated variants combined with *KRT25* genotypes detected in 148 curly coated and 68 straight coated horses is shown. Individuals, whose phenotypes could not be explained by these genotypic combinations, are printed in bold. Homozygous wild type (0/0), heterozygous (0/1) and homozygous mutant (1/1) genotypes are displayed.

	1/1-0/0	1/1-0/1	1/1-1/1	0/1-1/1	0/1-0/1	0/1-0/0	0/0-1/1	0/0-0/1	0/0-0/0
KRT25- ENSECAG00000014468 (NC_009154.2:g.21338314G>C)									
curly	23 (10.65)	0 (0.00)	0 (0.00)	0 (0.00)	9 (4.17)	90 (41.67)	4 (1.85)	22 (10.19)	0 (0.00)
straight	0 (0.00)	0 (0.00)	0 (0.00)	0 (0.00)	0 (0.00)	0 (0.00)	0 (0.00)	**2 (0.93)**	66 (30.56)
KRT25-KRTAP16 (NC_009154.2:g.21414219G>A)									
curly	23 (10.65)	0 (0.00)	0 (0.00)	0 (0.00)	8 (3.70)	91 (42.13)	4 (1.85)	22 (10.19)	0 (0.00)
straight	0 (0.00)	0 (0.00)	0 (0.00)	0 (0.00)	0 (0.00)	0 (0.00)	0 (0.00)	**1 (0.46)**	67 (31.02)
KRT25- TOP2A (NC_009154.2:g.22186465C>T)									
curly	4 (1.85)	11 (5.09)	8 (3.70)	0 (0.00)	62 (28.70)	37 (17.13)	0 (0.00)	0 (0.00)	**26 (12.04)**
straight	0 (0.00)	0 (0.00)	0 (0.00)	0 (0.00)	0 (0.00)	0 (0.00)	0 (0.00)	0 (0.00)	68 (31.48)
KRT25- SP6 (NC_009154.2:g.24022045C>T)									
curly	23 (10.65)	0 (0.00)	0 (0.00)	0 (0.00)	7 (3.24)	92 (42.59)	3 (1.39)	23 (10.65)	0 (0.00)
straight	0 (0.00)	0 (0.00)	0 (0.00)	0 (0.00)	0 (0.00)	0 (0.00)	0 (0.00)	0 (0.00)	68 (31.48)

KRT25-EPN3 (NC_009154.2:g.26187590C>T)									
curly	6 (2.78)	11 (5.09)	6 (2.78)	19 (8.80)	32 (14.81)	48 (22.22)	0 (0.00)	0 (0.00)	**26 (12.04)**
straight	0 (0.00)	0 (0.00)	0 (0.00)	0 (0.00)	0 (0.00)	0 (0.00)	0 (0.00)	**3 (1.39)**	65 (30.09)

Table S8. Phenotypes and mapping parameters for RNA sequencing. The number of reads and bases mapped in nine curly coated American Bashkir Curly Horses (ABCH), three straight coated ABCHs and three straight coated Quarter Horses is displayed.

Breed/population	Sex	Coat	Hypotrichosis	Reads mapped	Bases mapped
American Bashkir Curly Horse	Male	Curly	Complete	102754007	6529921571
American Bashkir Curly Horse	Male	Curly	Incomplete	72313103	4472671451
American Bashkir Curly Horse	Female	Curly	Incomplete	79700094	5060523139
American Bashkir Curly Horse	Male	Curly	Incomplete	52367893	3375086731
American Bashkir Curly Horse	Female	Curly	Incomplete	88947276	5687205346
American Bashkir Curly Horse	Female	Curly	Incomplete	76891720	4893573232
American Bashkir Curly Horse	Male	Curly	Incomplete	76317997	4841363406
American Bashkir Curly Horse	Male	Curly	Incomplete	67934330	4319000221
American Bashkir Curly Horse	Male	Curly	Not at all	83890511	5327321824
American Bashkir Curly Horse	Male	Straight	Not at all	86308198	5550654192
American Bashkir Curly Horse	Female	Straight	Not at all	100509078	6413309124
American Bashkir Curly Horse	Female	Straight	Not at all	58774286	3819752314
Quarter Horse	Female	Straight	Not at all	68276034	4374102057
Quarter Horse	Male	Straight	Not at all	68092476	4452927213
Quarter Horse	Female	Straight	Not at all	70727512	4338270428

Additional Data Table S9 (separate file)

Table S9. RNA-Seq results. This table displays the results from the differential expression analysis from the RNA-seq experiment to compare the expression profiles of straight and curly coated horses. Columns A-E provide different identifiers for the mRNA transcripts that were found in the RNA-seq experiment; columns F-H provide the chromosomal position of each gene; columns I-K provide the mean normalized expression levels of the whole group, the straight coated group (baseMeanA) and the curly coated group (baseMeanB); column M displays the log2FoldChange; column N displays the raw p-value from DESeq; the last column displays the p-values after FDR-adjustment.

Additional Data Table S10 (separate file)

Table S10. Validation results of differentially expressed genes derived from RNA-Seq. In total, 20 differentially expressed genes near *KRT25* and *SP6* known to be involved in hair development and harboring a significant p-value were validated in 38 horses and investigated for significant differences in mRNA expression in curly versus straight coated horses with regard to *KRT25* and *SP6* genotypes. Results from generalize mixed model analysis and the individual expression levels of all 38 tested samples are shown. In addition, *STAT5A* and *KRT25* were used as controls.

Table S11. Predicted protein interactions. Proteins and their corresponding horse orthologue genes potentially interacting with KRT25 and SP6 were identified using the BioGRID and IntAct database. Log2 fold changes and adjusted P-values detected in RNA-Seq analysis in the group comparisons curly versus straight, mutant KRT25 horses versus wild types as well as mutant SP6 versus wild types are shown.

**Group comparisons**					**Curly versus straight**		**Mutant KRT25 versus wild type KRT25**		**Mutant SP6 versus wild type SP6**	
**Interacting protein (human proteins)**	**Gene name (horse orthologue)**	**ECA**	**Start position**	**End position**	**log2 fold change**	**padj**	**log2 fold change**	**padj**	**log2 fold change**	**padj**
**KRT25 interactors**										
MORN4	MORN4	1	31680935	31683591	0.014976923	1	0.137724239	1	-0.13296354	1
CYLD	CYLD	3	3607812	3673138	-0.544508909	1	-0.339484652	1	-0.44867278	1
KRT83	ENSECAG00000015478 (KRT83)	6	69416432	69422664	0.673377362	1	0.587744236	1	-0.06211933	1
K2C72	KRT72	6	69680384	69692566	0.204299822	1	-0.448695707	1	1.11977739	1
K22e	KRT2	6	69733343	69740419	-3.250653446	0.131972389	-3.93220949	0.09709317	-1.43584772	1
K2C4	KRT4	6	69895499	69902053	-3.720319043	1	-3.328001621	1	-1.53334022	1
K2C3	KRT3	6	69875412	69881145	0.121134732	1	0.513452154	1	-Inf	1
K2C79	KRT79	6	69912416	69924148	-1.498499903	0.59517354	-3.700926561	0.01056836	0.69129835	1
K2C6C	KRT6C	6	69553390	69558280	0.194474777	1	-0.274933859	1	0.92431145	1
K2C75	KRT75	6	69523789	69533483	-1.254693772	0.550195846	-1.242634699	0.56985704	-1.04571421	1
K2C71	KRT71	6	69646737	69655337	0.099585112	1	-0.215488074	1	0.47822733	1

K22O	KRT76	6	69853986	69862262	1.369966575	1	1.762283998	1	-Inf	1
K2C1	KRT1	6	69772970	69796434	-1.980589307	0.019773987	-2.967986391	0.00040778	-0.18945088	1
K2C5	KRT5	6	69613165	69619519	-1.697868008	0.140791022	-1.760922168	0.14541938	-1.02042837	1
K2C6A	ENSECAG00000017229 (KRT6A)	6	69588835	69593753	-1.533214615	0.479358604	-1.958717984	0.19829148	-0.2530786	1
K2C6B	ENSECAG00000014899 (KRT6B)	6	69569389	69574549	-1.849601215	0.319568798	-2.655577472	0.06496675	-0.23122719	1
K2C8	ENSECAG00000021775 (KRT8)	6	69929954	69974726	-1.93746181	0.769248774	-1.73464747	0.90066115	-2.49883778	1
KRT86	ENSECAG00000009201 (KRT86)	6	69402662	69409182	0.503433207	1	0.499873475	1	-0.12710656	1
HGS	HGS	11	1471029	1484656	0.45975519	1	0.579067488	1	-0.0679603	1
PEPL	PPL	13	38132956	38154693	0.035780449	1	0.189653915	1	0.02440186	1
**SP6 interactors**										
SIN3A	SIN3A	1	118520678	118568520	-0.347585372	1	-0.12301196	1	-0.50116618	1
SORL1	SORL1	7	28934178	29071598	0.031382819	1	0.201341176	1	0.19193085	1
C18ORF8	C18ORF8	8	38480690	38501487	0.443213221	1	0.489465527	1	0.08115382	1
DNAAf3	ENSECAG00000000799 (DNAAf3)	10	24374034	24379289	-0.923375309	1	-0.531057886	1	-0.61153244	1
MYO18A	MYO18A	11	42937990	43021790	0.443214022	1	0.637452	1	-0.00246972	1
CCZ1	ENSECAG00000011387	13	1390361	1414560	0.059663966	1	0.126930827	1	0.03738984	1
TRAF7	TRAF7	13	40629939	40641344	0.437278195	1	0.459281318	1	-0.20641965	1
GNB2	GNB2	13	8678102	8680728	0.802092295	1	0.687066719	1	0.02542066	1
MON1A	MON1A	16	37261446	37275361	0.959248924	1	0.763141364	1	-0.14871375	1
EP300	EP300	28	37623209	37697145	0.090509885	1	0.344512038	1	-0.17986716	1

Table S12. Genotyping of significantly genome-wide associated SNPs. For subsequent chromosome-wide association analysis on ECA11, seven highly significant SNPs were genotyped in additional 139 samples using Kompetitive Allele Specific PCR (KASP). Bead chip ID, position, base change, forward and reverse primer, amplicon size (AS) and annealing temperature (AT) are given.

**ECA**	**Bead chip ID**	**Position**	**Base change**	**Forward primer(s) (5'-3')**	**Reverse primer (5'-3')**	**AS (bp)**	**AT (°C)/ cycles**
11	AX-104299273	21899031	G>A	GACGTCACCAACCAGAAACAGC-FAM<br>GACGTCACCAACCAGAAACAGT-VIC	CCAGATCTTCCTATGCTTAGTTCCTTTT	70	61/ 26
11	AX-104617737	21907825	G>A	AATCTATTCTCTCTGTGTTGAAGTCC-FAM<br>AATCTATTCTCTCTGTGTTGAAGTCT-VIC	GGGAATCAGTAAATGGTAAAGTATTCTGAA	66	61/ 32
11	AX-103834321	21910866	T>C	GCCAGCTACTGGTGCTTGTTCT-FAM<br>CCAGCTACTGGTGCTTGTTCC-VIC	CAATTGTATGATGTGGGCACTGATGAAT	51	61/ 26
11	AX-104641671	21922261	A>C	GTACATGAATGTTCATAGCAACATTATTCAT-FAM<br>ACATGAATGTTCATAGCAACATTATTCAG-VIC	GTGATGAACCTCTGGCTTATATACACTTT	69	61/ 26
11	AX-104311931	21999530	T>C	TTACTTTTAATTTTTATCAATTTACACATGTA-FAM<br>TACTTTTAATTTTTATCAATTTACACATGTG-VIC	AGTACATTTACGCACTGACAACAACTGTA	61	61/ 29
11	AX-103796906	22006773	A>G	ATTGATATTTTTTACAAGCTTTATGCATTCAT-FAM<br>ATTGATATTTTTTACAAGCTTTATGCATTCAC-VIC	CCCTTACTACTACAGATAGCCATGATTT	76	61/ 26
11	AX-104824172	35414844	T>G	CATACCCTATCATTTGCCACTCCTTA-FAM<br>ATACCCTATCATTTGCCACTCCTTC-VIC	AGGTTCCAAGTGGGCTTCCTCAAAA	51	61/ 32

Table S13. Results from filtering analysis of whole genome sequencing data. In total 386 variants on equine chromosome (ECA) 11 in the region of the keratin cluster proximal of the region of association and in the peak region of association (21,162,881-35,414,844 bp) could be exclusively found in one, two or all three curly coated horses. The chromosomal position, mutation type designated as SNP (S) or Indel (I), variant type, effect and affected gene are shown.

ECA	Position	Mutation type	Genotype Horse 1	Genotype Horse 2	Genotype Horse 3	Variant type	Effect	Gene	Mutation
11	21167798	S	0/1	0/1	0/0	intron variant	modifier	*KRT17*	c.1208-670G>C
11	21228085	S	0/0	0/1	0/0	intergenic region	modifier	*KRT14-ENSECAG00000014086*	n.21228085C>T
11	21338314	S	0/1	0/0	1/1	missense variant	moderate	*ENSECAG00000014468*	c.1034G>C, p.Gly345Ala
11	21340595	S	0/1	0/0	1/1	downstream gene variant	modifier	*ENSECAG00000014468*	c.*1340C>T
11	21343020	S	0/1	0/0	1/1	upstream gene variant	modifier	*ENSECAG00000015169*	c.-4071A>G
11	21344330	S	0/1	0/0	1/1	upstream gene variant	modifier	*ENSECAG00000015169*	c.-2761C>T
11	21348874	S	0/1	0/0	1/1	intron variant	modifier	*ENSECAG00000015169*	c.595-146C>T
11	21352402	S	0/1	0/0	1/1	downstream gene variant	modifier	*ENSECAG00000015169*	c.*1790G>A
11	21353935	S	0/1	0/0	1/1	downstream gene variant	modifier	*ENSECAG00000015169*	c.*3323G>C
11	21367930	S	0/1	0/0	0/1	upstream gene variant	modifier	*ENSECAG00000013448*	c.-1738C>T

11	21389828	S	0/1	0/0	1/1	downstream gene variant	modifier	*ENSECAG00000015178*	c.*1434A>T
11	21400197	S	0/1	0/0	1/1	intergenic region	modifier	*ENSECAG00000015178-KRTAP16-1*	n.21400197A>G
11	21401097	S	0/1	0/0	1/1	intergenic region	modifier	*ENSECAG00000015178-KRTAP16-1*	n.21401097G>C
11	21404074	S	0/1	0/0	1/1	intergenic region	modifier	*ENSECAG00000015178-KRTAP16-1*	n.21404074A>G
11	21405051	S	0/1	0/0	1/1	intergenic region	modifier	*ENSECAG00000015178-KRTAP16-1*	n.21405051C>G
11	21410759	S	0/1	0/0	1/1	upstream gene variant	modifier	*KRTAP16-1*	c.-2071C>G
11	21412015	S	0/1	0/0	1/1	upstream gene variant	modifier	*KRTAP16-1*	c.-815A>G
11	21412727	S	0/1	0/0	1/1	upstream gene variant	modifier	*KRTAP16-1*	c.-103C>T
11	21413753	S	0/1	0/0	1/1	synonymous variant	low	*KRTAP16-1*	c.603C>T, p.Ile201Ile
11	21413825	S	0/1	0/0	1/1	synonymous variant	low	*KRTAP16-1*	c.675T>A, p.Ser225Ser
11	21414219	S	0/1	0/0	1/1	missense variant	moderate	*KRTAP16-1*	c.1069G>A, p.Val357Ile
11	21416278	S	0/1	0/0	1/1	downstream gene variant	modifier	*KRTAP16-1*	c.*1628C>T

11	21419682	S	0/1	0/0	1/1	intergenic region	modifier	*KRTAP16-1-ENSECAG00000010427*	n.21419682G>A
11	21423179	S	0/1	0/0	1/1	intergenic region	modifier	*KRTAP16-1-ENSECAG00000010427*	n.21423179A>G
11	21423565	S	0/1	0/0	1/1	intergenic region	modifier	*KRTAP16-1-ENSECAG00000010427*	n.21423565C>G
11	21428656	S	0/0	0/0	1/1	intergenic region	modifier	*KRTAP16-1-ENSECAG00000010427*	n.21428656T>G
11	21429489	S	0/1	0/0	1/1	intergenic region	modifier	*KRTAP16-1-ENSECAG00000010427*	n.21429489A>G
11	21435656	S	0/1	0/1	0/0	intergenic region	modifier	*KRTAP16-1-ENSECAG00000010427*	n.21435656G>A
11	21436500	S	0/1	0/1	0/0	intergenic region	modifier	*KRTAP16-1-ENSECAG00000010427*	n.21436500G>A
11	21443253	S	0/1	0/1	0/0	intron variant	modifier	*ENSECAG00000010427*	c.411-885C>G
11	21444177	S	0/1	0/1	0/0	intron variant	modifier	*ENSECAG00000010427*	c.411-1809A>C
11	21449199	S	0/1	0/1	0/0	intron variant	modifier	*ENSECAG00000010427*	c.410+1228C>T
11	21449302	S	0/1	0/1	0/0	intron variant	modifier	*ENSECAG00000010427*	c.410+1125C>G
11	21449695	S	0/1	0/1	0/0	intron variant	modifier	*ENSECAG00000010427*	c.410+732A>G
11	21450215	S	0/1	0/1	0/0	intron variant	modifier	*ENSECAG00000010427*	c.410+212A>T

11	21450364	S	0/1	0/1	0/0	intron variant	modifier	*ENSECAG00000010427*	c.410+63A>C
11	21450870	S	0/1	0/1	0/0	intron variant	modifier	*ENSECAG00000010427*	c.361-394T>C
11	21458180	S	0/1	0/1	0/0	upstream gene variant	modifier	*ENSECAG00000010427*	c.-1662G>T
11	21460940	S	0/1	0/1	0/0	upstream gene variant	modifier	*ENSECAG00000010427*	c.-4422A>G
11	21463315	S	0/1	0/1	0/0	intergenic region	modifier	*ENSECAG00000010427-KRTAP4-1*	n.21463315G>A
11	21465184	S	0/1	0/1	0/0	intergenic region	modifier	*ENSECAG00000010427-KRTAP4-1*	n.21465184C>A
11	21465503	S	0/1	0/1	0/0	intergenic region	modifier	*ENSECAG00000010427-KRTAP4-1*	n.21465503G>T
11	21466033	I	0/1	0/1	0/0	intergenic region	modifier	*ENSECAG00000010427-KRTAP4-1*	n.21466034_21466037delTAAT
11	21509640	S	0/1	0/0	1/1	intergenic region	modifier	*KRTAP4-1-ENSECAG00000001948*	n.21509640T>C
11	21512931	S	0/1	0/0	1/1	intergenic region	modifier	*KRTAP4-1-ENSECAG00000001948*	n.21512931C>T
11	21517057	S	0/1	0/0	1/1	intergenic region	modifier	*KRTAP4-1-ENSECAG00000001948*	n.21517057C>T
11	21523731	S	0/1	0/0	1/1	intergenic region	modifier	*KRTAP4-1-ENSECAG00000001948*	n.21523731T>C
11	21526661	S	0/1	0/0	1/1	intergenic region	modifier	*KRTAP4-1-ENSECAG00000001948*	n.21526661A>G

11	21527856	S	0/1	0/0	1/1	intergenic region	modifier	*KRTAP4-1-ENSECAG00000001948*	n.21527856A>C
11	21529220	I	0/1	0/0	1/1	intergenic region	modifier	*KRTAP4-1-ENSECAG00000001948*	n.21529221_21529237delACTTTCTCTGGCTGAAG
11	21531232	S	0/1	0/0	1/1	intergenic region	modifier	*KRTAP4-1-ENSECAG00000001948*	n.21531232C>T
11	21568392	S	0/1	0/0	1/1	intergenic region	modifier	*KRTAP4-1-ENSECAG00000001948*	n.21568392G>A
11	21705617	S	0/1	0/1	0/0	intron variant	modifier	*KRT23*	c.387+53C>G
11	21731185	S	0/1	0/0	1/1	intergenic region	modifier	*KRT23-KRT20*	n.21731185G>A
11	21732868	S	0/1	0/0	1/1	intergenic region	modifier	*KRT23-KRT20*	n.21732868G>C
11	21738151	S	0/1	0/0	1/1	intergenic region	modifier	*KRT23-KRT20*	n.21738151C>T
11	21741074	S	0/1	0/0	1/1	intergenic region	modifier	*KRT23-KRT20*	n.21741074A>G
11	21774363	S	0/1	0/0	1/1	downstream gene variant	modifier	*KRT12*	c.*4878A>G
11	21774764	S	0/1	0/0	1/1	intergenic region	modifier	*KRT12-ENSECAG00000020036*	n.21774764G>A
11	21777773	S	0/1	0/0	1/1	intergenic region	modifier	*KRT12-ENSECAG00000020036*	n.21777773C>G
11	21778160	S	0/1	0/0	1/1	intergenic region	modifier	*KRT12-ENSECAG00000020036*	n.21778160C>G

11	21783338	S	0/1	0/0	1/1	intergenic region	modifier	*KRT12-ENSECAG00000020036*	n.21783338C>A
11	21784690	S	0/1	0/0	1/1	intergenic region	modifier	*KRT12-ENSECAG00000020036*	n.21784690T>A
11	21785297	S	0/1	0/0	1/1	intergenic region	modifier	*KRT12-ENSECAG00000020036*	n.21785297C>T
11	21786431	S	1/1	0/0	1/1	intergenic region	modifier	*KRT12-ENSECAG00000020036*	n.21786431G>A
11	21787307	S	0/1	./.	0/1	intergenic region	modifier	*KRT12-ENSECAG00000020036*	n.21787307C>A
11	21818502	I	0/1	0/0	0/1	upstream gene variant	modifier	*ENSECAG00000020036*	c.-765_-764insT
11	21823805	S	0/1	0/0	0/1	intron variant	modifier	*ENSECAG00000020036*	c.*211+1022A>G
11	21827062	S	0/1	0/0	0/1	intron variant	modifier	*ENSECAG00000020036*	c.*211+4279G>A
11	21830964	S	1/1	0/0	1/1	upstream gene variant	modifier	*ENSECAG00000024656*	c.-4169T>C
11	21839860	S	0/1	0/0	0/1	downstream gene variant	modifier	*ENSECAG00000020036*	c.*17724A>G
11	21843275	S	0/1	0/0	0/1	upstream gene variant	modifier	*KRT28*	c.-3997G>A
11	21849036	S	0/1	0/0	1/1	intron variant	modifier	*KRT28*	c.690+68A>G

11	21852806	S	0/1	0/0	1/1	intron variant	modifier	*KRT28*	c.978+1419C>T
11	21856065	S	0/1	0/0	1/1	downstream gene variant	modifier	*KRT28*	c.*415A>C
11	21872286	S	0/1	0/0	1/1	downstream gene variant	modifier	*KRT27*	c.*949C>T
11	21889206	S	0/1	0/0	1/1	upstream gene variant	modifier	*KRT25*	c.-1689A>G
11	21891160	S	0/1	1/1	0/0	missense variant	moderate	*KRT25*	c.266G>A, p.Arg89His
11	21891791	S	0/1	0/0	0/1	intron variant	modifier	*KRT25*	c.512+75C>A
11	21893617	S	0/1	0/0	0/1	intron variant	modifier	*KRT25*	c.670-851A>C
11	21940407	S	0/1	0/0	1/1	downstream gene variant	modifier	*KRT24*	c.*4316A>G
11	22186465	S	0/1	0/1	0/0	missense variant	moderate	*TOP2A*	c.3263C>T, p.Ser1088Leu
11	22239059	S	0/0	0/1	0/0	intron variant	modifier	*RARA*	c.-365-545A>G
11	22552240	S	0/1	0/1	0/0	intergenic region	modifier	*GSDMB-ZPBP2*	n.22552240A>G
11	22617510	S	0/1	0/0	1/1	intron variant	modifier	*IKZF3*	c.-8-13684C>G
11	22689375	S	0/1	0/1	0/0	intron variant	modifier	*ERBB2*	c.1955+546G>A
11	22827170	S	0/1	0/1	0/0	intergenic region	modifier	*NEUROD2-CDK12*	n.22827170T>C
11	22827171	S	0/1	0/1	0/0	intergenic region	modifier	*NEUROD2-CDK12*	n.22827171T>G

11	22852158	S	0/1	0/1	0/0	intron variant	modifier	*CDK12*	c.3013+370A>G
11	22874497	S	0/1	0/1	0/0	intron variant	modifier	*CDK12*	c.1185-1646A>G
11	23084469	S	0/1	1/1	0/0	intron variant	modifier	*CACNB1*	c.899-15C>T
11	23095844	S	0/1	0/1	0/0	upstream gene variant	modifier	*ARL5C*	c.-427C>T
11	23330268	S	0/1	0/0	1/1	upstream gene variant	modifier	*PIP4K2B*	c.-4549A>G
11	23341241	S	0/1	0/1	0/0	intron variant	modifier	*PIP4K2B*	c.349-95G>A
11	23342164	S	0/1	0/1	0/0	intron variant	modifier	*PIP4K2B*	c.496-342C>T
11	23471138	S	0/0	0/1	0/0	intergenic region	modifier	*MLLT6-SRCIN1*	n.23471138T>C
11	23487347	S	0/0	0/1	0/0	intron variant	modifier	*SRCIN1*	c.124+8699A>G
11	23579187	S	0/1	0/0	1/1	intergenic region	modifier	*ENSECAG00000021431-SOCS7*	n.23579187G>A
11	23649377	S	0/1	0/0	1/1	intron variant	modifier	*SOCS7*	c.*21-419G>A
11	23764470	S	0/1	0/1	0/0	intergenic region	modifier	*ENSECAG00000000590-NPEPPS*	n.23764470T>A
11	23797288	S	0/1	0/0	1/1	intron variant	modifier	*NPEPPS*	c.310-587G>C
11	23801182	S	0/1	0/1	0/0	intron variant	modifier	*NPEPPS*	c.757-713C>T
11	23840411	S	0/1	0/1	0/0	intergenic region	modifier	*NPEPPS-KPNB1*	n.23840411C>T

11	23852906	S	0/0	0/1	0/0	upstream gene variant	modifier	*KPNB1*	c.-648G>A
11	23904818	S	0/1	0/1	0/0	downstream gene variant	modifier	*TBKBP1*	c.*1216G>T
11	23973360	S	0/1	0/0	1/1	intergenic region	modifier	*ENSECAG00000018160-OSBPL7*	n.23973360A>G
11	23973460	S	0/1	0/0	1/1	intergenic region	modifier	*ENSECAG00000018160-OSBPL7*	n.23973460G>A
11	23973540	S	0/1	0/0	1/1	intergenic region	modifier	*ENSECAG00000018160-OSBPL7*	n.23973540A>G
11	23973775	S	0/1	0/0	1/1	intergenic region	modifier	*ENSECAG00000018160-OSBPL7*	n.23973775T>C
11	23975325	S	0/1	0/0	1/1	intergenic region	modifier	*ENSECAG00000018160-OSBPL7*	n.23975325C>T
11	23975461	S	0/1	0/0	1/1	intergenic region	modifier	*ENSECAG00000018160-OSBPL7*	n.23975461T>G
11	23975678	S	0/1	0/0	1/1	intergenic region	modifier	*ENSECAG00000018160-OSBPL7*	n.23975678G>A
11	23975959	S	0/1	0/0	1/1	intergenic region	modifier	*ENSECAG00000018160-OSBPL7*	n.23975959T>C
11	23976172	S	0/1	0/0	1/1	intergenic region	modifier	*ENSECAG00000018160-OSBPL7*	n.23976172A>G
11	23976788	S	0/1	0/0	1/1	intergenic region	modifier	*ENSECAG00000018160-OSBPL7*	n.23976788A>G
11	23977213	S	0/1	0/0	1/1	intergenic region	modifier	*ENSECAG00000018160-OSBPL7*	n.23977213G>A
11	23977755	S	0/1	0/0	1/1	intergenic region	modifier	*ENSECAG00000018160-OSBPL7*	n.23977755T>C
11	23977883	S	0/1	0/0	1/1	intergenic region	modifier	*ENSECAG00000018160-OSBPL7*	n.23977883C>T
11	23977929	S	0/1	0/0	1/1	intergenic region	modifier	*ENSECAG00000018160-OSBPL7*	n.23977929G>C

11	23978368	S	0/1	0/0	1/1	intergenic region	modifier	*ENSECAG00000018160-OSBPL7*	n.23978368C>T
11	23979549	S	0/1	0/0	1/1	intergenic region	modifier	*ENSECAG00000018160-OSBPL7*	n.23979549C>T
11	23979653	I	0/1	0/0	1/1	intergenic region	modifier	*ENSECAG00000018160-OSBPL7*	n.23979654_23979657delAGGG
11	23979720	S	0/1	0/0	1/1	intergenic region	modifier	*ENSECAG00000018160-OSBPL7*	n.23979720G>A
11	23979757	S	0/1	0/0	1/1	intergenic region	modifier	*ENSECAG00000018160-OSBPL7*	n.23979757C>T
11	23980255	S	0/1	0/0	1/1	intergenic region	modifier	*ENSECAG00000018160-OSBPL7*	n.23980255G>A
11	23980574	S	1/1	0/0	1/1	intergenic region	modifier	*ENSECAG00000018160-OSBPL7*	n.23980574C>T
11	23981287	S	0/1	0/0	1/1	downstream gene variant	modifier	*OSBPL7*	c.*4878C>T
11	23981642	S	0/1	0/0	1/1	downstream gene variant	modifier	*OSBPL7*	c.*4523G>A
11	23981650	S	0/1	0/0	1/1	downstream gene variant	modifier	*OSBPL7*	c.*4515A>C
11	23981909	S	0/1	0/0	1/1	downstream gene variant	modifier	*OSBPL7*	c.*4256C>G
11	23982327	S	0/1	0/0	1/1	downstream gene variant	modifier	*OSBPL7*	c.*3838C>T
11	23982610	S	0/1	0/0	1/1	downstream gene variant	modifier	*OSBPL7*	c.*3555C>T

11	23982805	S	1/1	0/0	1/1	downstream gene variant	modifier	*OSBPL7*	c.*3360C>T
11	23982896	S	0/1	0/0	1/1	downstream gene variant	modifier	*OSBPL7*	c.*3269G>C
11	23982923	I	0/1	0/0	1/1	downstream gene variant	modifier	*OSBPL7*	c.*3241_*3242insGG
11	23983117	S	0/1	0/0	1/1	downstream gene variant	modifier	*OSBPL7*	c.*3048T>C
11	23983449	S	0/1	0/0	1/1	downstream gene variant	modifier	*OSBPL7*	c.*2716T>C
11	23983817	S	0/1	0/0	1/1	downstream gene variant	modifier	*OSBPL7*	c.*2348T>A
11	23983862	S	0/1	0/0	1/1	downstream gene variant	modifier	*OSBPL7*	c.*2303G>T
11	23983908	I	0/1	0/0	1/1	downstream gene variant	modifier	*OSBPL7*	c.*2254_*2256delCTT
11	23983916	S	0/1	0/0	1/1	downstream gene variant	modifier	*OSBPL7*	c.*2249G>A
11	23984392	S	0/1	0/0	1/1	downstream gene variant	modifier	*OSBPL7*	c.*1773G>A
11	23984524	S	0/1	0/0	1/1	downstream gene variant	modifier	*OSBPL7*	c.*1641T>C

11	23984627	S	0/1	0/0	1/1	downstream gene variant	modifier	*OSBPL7*	c.*1538A>T
11	23984681	S	0/1	0/0	1/1	downstream gene variant	modifier	*OSBPL7*	c.*1484C>T
11	23984687	S	0/1	0/0	1/1	downstream gene variant	modifier	*OSBPL7*	c.*1478C>T
11	23984781	S	0/1	0/0	1/1	downstream gene variant	modifier	*OSBPL7*	c.*1384T>A
11	23984819	S	0/1	0/0	1/1	downstream gene variant	modifier	*OSBPL7*	c.*1346G>A
11	23984884	S	0/1	0/0	1/1	downstream gene variant	modifier	*OSBPL7*	c.*1281C>G
11	23985801	S	0/1	0/0	1/1	3 prime UTR variant	modifier	*OSBPL7*	c.*364C>T
11	23986276	S	0/1	0/0	1/1	splice region variant&intron variant	low	*OSBPL7*	c.2421-3C>T
11	23986507	S	0/1	0/0	1/1	synonymous variant	low	*OSBPL7*	c.2325C>T, p.Ala775Ala
11	23986655	S	0/1	0/0	1/1	intron variant	modifier	*OSBPL7*	c.2297+88C>G
11	23986865	S	0/1	0/0	1/1	synonymous variant	low	*OSBPL7*	c.2175G>C, p.Ser725Ser
11	23994766	S	0/1	0/0	1/1	intron variant	modifier	*OSBPL7*	c.703-168C>T

11	24005125	S	0/1	0/0	1/1	upstream gene variant	modifier	*LRRC46*	c.-1699G>A
11	24006223	S	0/1	0/0	1/1	synonymous variant	low	*MRPL10*	c.33G>C, p.Gly11Gly
11	24006490	S	0/1	0/0	1/1	upstream gene variant	modifier	*MRPL10*	c.-235C>G
11	24008198	S	0/1	0/0	1/1	upstream gene variant	modifier	*MRPL10*	c.-1943G>A
11	24008226	S	0/1	0/0	1/1	upstream gene variant	modifier	*MRPL10*	c.-1971G>A
11	24008302	S	0/1	0/0	1/1	upstream gene variant	modifier	*MRPL10*	c.-2047A>T
11	24009269	S	0/1	0/0	1/1	upstream gene variant	modifier	*MRPL10*	c.-3014A>T
11	24013169	S	0/1	0/0	1/1	downstream gene variant	modifier	*LRRC46*	c.*1724T>G
11	24022045	S	0/1	0/0	1/1	missense variant	moderate	*SP6*	c.1090G>A, p.Gly364Ser
11	24023489	S	0/1	0/1	0/0	intron variant	modifier	*SP6*	c.-58-297G>A
11	24023520	S	0/1	0/0	1/1	intron variant	modifier	*SP6*	c.-58-328G>C
11	24042899	S	0/1	0/0	1/1	intergenic region	modifier	*SP6-SP2*	n.24042899C>A
11	24042947	S	0/1	0/0	1/1	intergenic region	modifier	*SP6-SP2*	n.24042947G>C

11	24059501	S	0/1	0/0	1/1	intergenic region	modifier	*SP6-SP2*	n.24059501G>A
11	24060495	S	0/1	0/0	1/1	intergenic region	modifier	*SP6-SP2*	n.24060495G>A
11	24060627	S	0/1	0/0	1/1	intergenic region	modifier	*SP6-SP2*	n.24060627G>A
11	24061863	S	0/1	0/0	1/1	intergenic region	modifier	*SP6-SP2*	n.24061863G>T
11	24061927	S	0/1	0/0	1/1	intergenic region	modifier	*SP6-SP2*	n.24061927T>G
11	24062063	S	0/1	0/0	1/1	intergenic region	modifier	*SP6-SP2*	n.24062063T>C
11	24063677	S	0/1	0/0	1/1	intergenic region	modifier	*SP6-SP2*	n.24063677C>T
11	24064405	S	0/1	0/0	1/1	intergenic region	modifier	*SP6-SP2*	n.24064405G>A
11	24070645	S	0/1	0/0	1/1	intergenic region	modifier	*SP6-SP2*	n.24070645G>C
11	24071230	S	0/1	0/0	1/1	intergenic region	modifier	*SP6-SP2*	n.24071230G>A
11	24099059	S	0/1	0/0	1/1	intron variant	modifier	*PNPO*	c.273-253T>C
11	24117699	S	0/1	0/0	1/1	intergenic region	modifier	*PRR15L-CDK5RAP3*	n.24117699C>T
11	24120238	S	0/1	0/0	1/1	upstream gene variant	modifier	*CDK5RAP3*	c.-3412A>G
11	24121529	I	0/1	0/0	1/1	upstream gene variant	modifier	*CDK5RAP3*	c.-2120delG
11	24121531	S	0/0	0/0	1/1	upstream gene variant	modifier	*CDK5RAP3*	c.-2119G>T

11	24167645	S	0/1	0/1	0/0	intron variant	modifier	*COPZ2*	c.435+148C>T
11	24502900	S	0/1	0/1	0/0	intergenic region	modifier	*ENSECAG00000010544-HOXB1*	n.24502900A>G
11	24579880	S	0/1	0/1	0/0	downstream gene variant	modifier	*HOXB1*	c.*1945C>T
11	24678796	S	0/1	0/0	1/1	downstream gene variant	modifier	*eca-mir-196a*	n.*2829G>A
11	24914849	S	0/1	0/0	1/1	downstream gene variant	modifier	*UBE2Z*	c.*2293C>G
11	25005508	S	0/1	0/1	0/0	intergenic region	modifier	*IGF2BP1-ENSECAG00000013064*	n.25005508G>A
11	25218337	S	0/1	0/1	0/0	synonymous variant	low	*ZNF652*	c.246G>A, p.Arg82Arg
11	25335108	S	0/1	0/1	0/0	intron variant	modifier	*NXPH3*	c.29-85962C>T
11	25359112	S	0/1	0/0	1/1	upstream gene variant	modifier	*NGFR*	c.-4116T>C
11	25690046	S	0/0	0/1	0/0	intron variant	modifier	*FAM117A*	c.118-35308G>T
11	25833347	S	0/1	0/1	0/0	intron variant	modifier	*FAM117A*	c.34-34133G>A
11	26057603	S	0/0	0/1	0/0	downstream gene variant	modifier	*XYLT2*	c.*599G>C
11	26092383	S	0/1	0/1	0/0	upstream gene variant	modifier	*LRRC59*	c.-2666G>A

11	26111198	S	0/1	0/0	1/1	intron variant	modifier	*ACSF2*	c.134+1120A>G
11	26187590	S	0/1	0/1	0/0	missense variant	moderate	*EPN3*	c.104C>T, p.Pro35Leu
11	26290201	S	0/1	0/0	1/1	intron variant	modifier	*ABCC3*	c.61-3782G>A
11	26471559	S	0/1	0/0	1/1	intergenic region	modifier	*TOB1-SPAG9*	n.26471559G>A
11	26507057	S	0/1	0/1	0/0	intergenic region	modifier	*TOB1-SPAG9*	n.26507057G>C
11	26530496	S	0/1	0/1	0/0	intergenic region	modifier	*TOB1-SPAG9*	n.26530496G>A
11	26818187	S	0/0	0/1	0/0	intergenic region	modifier	*UTP18-CA10*	n.26818187C>T
11	26933902	S	0/1	0/1	0/0	intron variant	modifier	*CA10*	c.*269-45285G>A
11	27064572	S	0/1	0/0	1/1	intron variant	modifier	*CA10*	c.*268+5322A>G
11	27238367	I	0/1	0/0	1/1	intron variant	modifier	*CA10*	c.280-72245delA
11	27261936	S	0/1	0/1	0/0	intron variant	modifier	*CA10*	c.279+82749G>A
11	27287805	S	0/1	0/0	1/1	intron variant	modifier	*CA10*	c.279+56880T>C
11	27378717	S	0/1	0/1	0/0	intron variant	modifier	*CA10*	c.137-33890T>C
11	27465553	S	0/1	0/1	0/0	intron variant	modifier	*CA10*	c.62-8220A>T
11	27725425	S	0/1	0/1	0/0	intergenic region	modifier	*CA10-U3*	n.27725425G>A
11	27871612	S	0/1	0/0	1/1	intergenic region	modifier	*U3-ENSECAG00000004799*	n.27871612C>T

11	27889105	S	0/1	0/1	0/0	intergenic region	modifier	*U3-ENSECAG00000004799*	n.27889105G>T
11	27932277	S	0/1	0/1	0/0	intergenic region	modifier	*U3-ENSECAG00000004799*	n.27932277G>A
11	27984825	S	0/1	0/0	1/1	intergenic region	modifier	*U3-ENSECAG00000004799*	n.27984825G>C
11	28041238	S	0/1	0/1	0/0	intergenic region	modifier	*U3-ENSECAG00000004799*	n.28041238T>C
11	28395347	S	0/1	0/0	1/1	intergenic region	modifier	*U3-ENSECAG00000004799*	n.28395347C>T
11	28538084	S	0/0	0/1	0/0	intergenic region	modifier	*U3-ENSECAG00000004799*	n.28538084T>C
11	28565551	S	0/1	0/0	1/1	intergenic region	modifier	*U3-ENSECAG00000004799*	n.28565551C>T
11	28603533	S	0/1	0/0	1/1	intergenic region	modifier	*U3-ENSECAG00000004799*	n.28603533A>G
11	28777446	S	0/1	0/0	1/1	intergenic region	modifier	*ENSECAG00000004853-KIF2B*	n.28777446C>T
11	29324776	S	0/1	0/0	1/1	intergenic region	modifier	*ENSECAG00000014167-ENSECAG00000004877*	n.29324776A>G
11	29424752	S	0/1	0/0	1/1	intergenic region	modifier	*ENSECAG00000004877-ENSECAG00000014174*	n.29424752T>G

11	29436382	S	0/1	0/1	0/0	intergenic region	modifier	*ENSECAG00000004877-ENSECAG00000014174*	n.29436382A>G
11	29820448	S	0/1	0/0	1/1	intergenic region	modifier	*STXBP4-HLF*	n.29820448T>A
11	29820449	S	0/1	0/0	1/1	intergenic region	modifier	*STXBP4-HLF*	n.29820449A>G
11	29863174	I	0/1	0/0	1/1	intergenic region	modifier	*STXBP4-HLF*	n.29863175delA
11	29970918	I	0/1	0/0	1/1	intron variant	modifier	*HLF*	c.337+16431delC
11	30050817	S	0/1	0/1	0/0	downstream gene variant	modifier	*MMD*	c.*638T>C
11	30228332	S	0/1	0/0	1/1	intergenic region	modifier	*MMD-TMEM100*	n.30228332G>A
11	30264003	S	0/1	0/0	1/1	intergenic region	modifier	*MMD-TMEM100*	n.30264003C>A
11	30741613	S	0/1	0/1	0/0	intron variant	modifier	*ANKFN1*	c.22-18771G>A
11	31595433	S	0/0	0/1	0/0	intergenic region	modifier	*ENSECAG00000002926-MSI2*	n.31595433G>A
11	31829141	S	0/1	0/0	1/1	intron variant	modifier	*MSI2*	c.255+51810T>C
11	32023964	S	0/1	0/1	0/0	intron variant	modifier	*MSI2*	c.641-2299G>A
11	32104962	S	0/1	0/0	1/1	intergenic region	modifier	*CCDC182-MRPS23*	n.32104962A>G
11	32217771	S	0/1	0/0	1/1	intron variant	modifier	*CUEDC1*	c.-26+1776C>G
11	32325249	S	0/0	0/1	0/0	intron variant	modifier	*DYNLL2*	c.-16-35461C>T

11	32330280	S	0/0	0/1	0/0	intron variant	modifier	*DYNLL2*	c.-16-30430C>T
11	32335139	S	0/0	0/1	0/0	intron variant	modifier	*DYNLL2*	c.-16-25571G>A
11	32335702	S	0/0	0/1	0/0	intron variant	modifier	*DYNLL2*	c.-16-25008A>G
11	32338909	S	0/0	0/1	0/0	intron variant	modifier	*DYNLL2*	c.-16-21801G>A
11	32350911	S	0/0	0/1	0/0	intron variant	modifier	*DYNLL2*	c.-16-9799T>C
11	32352638	S	0/0	0/1	0/0	intron variant	modifier	*DYNLL2*	c.-16-8072A>T
11	32353521	S	0/0	0/1	0/0	intron variant	modifier	*DYNLL2*	c.-16-7189G>A
11	32353778	S	0/0	0/1	0/0	intron variant	modifier	*DYNLL2*	c.-16-6932T>C
11	32354109	S	0/1	0/1	0/0	intron variant	modifier	*DYNLL2*	c.-16-6601C>T
11	32356953	S	0/0	0/1	0/0	intron variant	modifier	*DYNLL2*	c.-16-3757G>A
11	32357167	S	0/0	0/1	0/0	intron variant	modifier	*DYNLL2*	c.-16-3543C>T
11	32358650	S	0/0	0/1	0/0	intron variant	modifier	*DYNLL2*	c.-16-2060G>A
11	32359300	S	0/0	0/1	0/0	intron variant	modifier	*DYNLL2*	c.-16-1410G>C
11	32360060	S	0/0	0/1	0/0	intron variant	modifier	*DYNLL2*	c.-16-650G>A
11	32361159	S	0/0	0/1	0/0	intron variant	modifier	*DYNLL2*	c.132+302G>A
11	32361290	I	0/0	0/1	0/0	intron variant	modifier	*DYNLL2*	c.132+433_132+434insA

11	32367532	S	0/0	0/1	0/0	downstream gene variant	modifier	*DYNLL2*	c.*4565A>G
11	32369138	S	0/0	0/1	0/0	intergenic region	modifier	*DYNLL2-OR4D1*	n.32369138C>T
11	32371695	S	0/0	0/1	0/0	intergenic region	modifier	*DYNLL2-OR4D1*	n.32371695C>T
11	32376870	S	0/0	0/1	0/0	intergenic region	modifier	*DYNLL2-OR4D1*	n.32376870G>A
11	32378173	S	0/0	0/1	0/0	intergenic region	modifier	*DYNLL2-OR4D1*	n.32378173G>A
11	32394342	S	0/0	0/1	0/0	intergenic region	modifier	*DYNLL2-OR4D1*	n.32394342A>G
11	32399970	S	0/0	0/1	0/0	intergenic region	modifier	*DYNLL2-OR4D1*	n.32399970C>G
11	32406645	S	0/0	0/1	0/0	intergenic region	modifier	*DYNLL2-OR4D1*	n.32406645A>G
11	32412894	S	0/1	0/1	0/0	downstream gene variant	modifier	*OR4D1*	c.*296G>C
11	32417371	S	0/0	0/1	0/0	downstream gene variant	modifier	*OR4D1*	c.*4773A>G
11	32538095	S	0/0	0/1	0/0	downstream gene variant	modifier	*LPO*	c.*4823A>C
11	32607419	S	0/1	0/1	0/0	upstream gene variant	modifier	*SUPT4H1*	c.-4257C>T
11	32809689	S	0/0	0/1	0/0	intron variant	modifier	*TEX14*	c.1209-3346G>T
11	32834006	S	0/1	0/1	0/0	downstream gene variant	modifier	*U3*	n.*356T>A

11	32836844	S	0/1	0/1	0/0	upstream gene variant	modifier	*U3*	n.-2269C>T
11	32893856	S	0/0	0/1	0/0	upstream gene variant	modifier	*U1*	n.-233A>G
11	32903120	I	0/0	0/1	0/0	intron variant	modifier	*RAD51C*	c.124+452delC
11	33039449	S	0/1	0/1	0/0	upstream gene variant	modifier	*ENSECAG00000003590*	c.-3564T>C
11	33091549	S	0/0	0/0	0/1	intron variant	modifier	*PPM1E*	c.-1-27809A>G
11	33099784	I	0/1	0/1	0/0	intron variant	modifier	*PPM1E*	c.-1-19573_-1-19570delTTGA
11	33204766	S	0/0	0/1	0/0	intron variant	modifier	*TRIM37*	c.1533+254T>A
11	33215757	S	0/0	0/1	0/0	intron variant	modifier	*TRIM37*	c.1019+5726A>C
11	33252841	S	0/0	0/1	0/0	upstream gene variant	modifier	*U6*	n.-3560G>A
11	33355082	S	0/1	0/0	1/1	intron variant	modifier	*GDPD1*	c.185+3479T>G
11	33366280	I	0/0	0/1	0/0	intron variant	modifier	*GDPD1*	c.486+106_486+107insA
11	33447756	S	0/1	0/1	0/0	intron variant	modifier	*YPEL2*	c.117+9544G>A
11	33601940	S	0/1	0/0	1/1	intergenic region	modifier	*ENSECAG00000018590-ENSECAG00000018606*	n.33601940G>A
11	33998716	I	0/1	0/1	0/0	intergenic region	modifier	*ENSECAG00000017636-MED13*	n.33998716_33998717insT

11	33999110	S	0/1	0/1	0/0	intergenic region	modifier	*ENSECAG00000017636-MED13*	n.33999110T>C
11	33999333	S	0/1	0/1	0/0	intergenic region	modifier	*ENSECAG00000017636-MED13*	n.33999333A>T
11	34002018	S	0/1	0/1	0/0	intergenic region	modifier	*ENSECAG00000017636-MED13*	n.34002018A>C
11	34004149	S	0/1	0/1	0/0	intergenic region	modifier	*ENSECAG00000017636-MED13*	n.34004149A>G
11	34004829	S	0/1	0/1	0/0	intergenic region	modifier	*ENSECAG00000017636-MED13*	n.34004829C>T
11	34013400	S	0/1	0/1	0/0	intergenic region	modifier	*ENSECAG00000017636-MED13*	n.34013400T>C
11	34015333	S	0/1	0/1	0/0	intergenic region	modifier	*ENSECAG00000017636-MED13*	n.34015333T>C
11	34015941	S	0/1	0/1	0/0	intergenic region	modifier	*ENSECAG00000017636-MED13*	n.34015941G>A
11	34019793	S	0/1	0/1	0/0	intergenic region	modifier	*ENSECAG00000017636-MED13*	n.34019793A>G
11	34020636	S	0/1	0/1	0/0	intergenic region	modifier	*ENSECAG00000017636-MED13*	n.34020636C>T
11	34029721	S	0/1	0/1	0/0	intergenic region	modifier	*ENSECAG00000017636-MED13*	n.34029721C>A
11	34034452	S	0/1	0/1	0/0	intergenic region	modifier	*ENSECAG00000017636-MED13*	n.34034452G>A
11	34040509	S	0/1	0/1	0/0	intergenic region	modifier	*ENSECAG00000017636-MED13*	n.34040509T>G
11	34049714	S	0/1	0/1	0/0	intergenic region	modifier	*ENSECAG00000017636-MED13*	n.34049714G>T
11	34061646	S	0/1	0/1	0/0	intergenic region	modifier	*ENSECAG00000017636-MED13*	n.34061646A>C
11	34091166	S	0/1	0/1	0/0	intergenic region	modifier	*ENSECAG00000017636-MED13*	n.34091166A>G
11	34091186	S	0/1	0/1	0/0	intergenic region	modifier	*ENSECAG00000017636-MED13*	n.34091186G>T

11	34094409	S	0/1	0/1	0/0	intergenic region	modifier	*ENSECAG00000017636-MED13*	n.34094409A>G
11	34103845	S	0/1	1/1	0/0	intergenic region	modifier	*ENSECAG00000017636-MED13*	n.34103845T>C
11	34118081	S	0/1	0/1	0/0	upstream gene variant	modifier	*MED13*	c.-4059A>G
11	34183990	S	0/0	0/1	0/0	intron variant	modifier	*MED13*	c.2823-154A>G
11	34255189	S	0/0	0/1	0/0	intron variant	modifier	*INTS2*	c.2008+3170T>A
11	34289462	S	0/0	0/1	0/0	intron variant	modifier	*BRIP1*	c.382+2933A>G
11	34295012	S	0/0	0/1	0/0	intron variant	modifier	*BRIP1*	c.630+391C>G
11	34314554	S	0/0	0/1	0/0	intron variant	modifier	*BRIP1*	c.630+19933G>A
11	34338610	S	0/0	0/1	0/0	intron variant	modifier	*BRIP1*	c.631-465A>G
11	34342206	S	0/0	0/1	0/0	intron variant	modifier	*BRIP1*	c.922-2357G>A
11	34349464	S	0/0	0/0	0/1	intron variant	modifier	*BRIP1*	c.1341-1329C>T
11	34349465	S	0/0	0/0	0/1	intron variant	modifier	*BRIP1*	c.1341-1328A>G
11	34349473	S	0/0	0/0	0/1	intron variant	modifier	*BRIP1*	c.1341-1320T>C
11	34349474	S	0/0	0/0	0/1	intron variant	modifier	*BRIP1*	c.1341-1319A>G
11	34349479	S	0/0	0/0	0/1	intron variant	modifier	*BRIP1*	c.1341-1314G>C
11	34349485	S	0/0	0/0	0/1	intron variant	modifier	*BRIP1*	c.1341-1308A>C

11	34349496	S	0/0	0/0	0/1	intron variant	modifier	*BRIP1*	c.1341-1297T>A
11	34349497	S	0/0	0/0	0/1	intron variant	modifier	*BRIP1*	c.1341-1296G>A
11	34349498	S	0/0	0/0	0/1	intron variant	modifier	*BRIP1*	c.1341-1295A>G
11	34349507	S	0/0	0/0	0/1	intron variant	modifier	*BRIP1*	c.1341-1286C>T
11	34351390	S	0/0	0/1	0/0	intron variant	modifier	*BRIP1*	c.1473+465A>G
11	34367709	S	0/0	0/1	0/0	intron variant	modifier	*BRIP1*	c.2094+1860C>T
11	34382128	S	0/0	0/1	0/0	intron variant	modifier	*BRIP1*	c.2094+16279T>C
11	34423150	S	0/0	0/1	0/0	downstream gene variant	modifier	*BRIP1*	c.*3529G>A
11	34424690	S	0/0	0/1	0/0	intergenic region	modifier	*BRIP1-TBX4*	n.34424690G>T
11	34454800	S	0/0	0/1	0/0	intergenic region	modifier	*BRIP1-TBX4*	n.34454800A>G
11	34459067	S	0/0	0/1	0/0	intergenic region	modifier	*BRIP1-TBX4*	n.34459067A>G
11	34459649	S	0/0	0/1	0/0	intergenic region	modifier	*BRIP1-TBX4*	n.34459649C>T
11	34460131	S	0/0	0/1	0/0	intergenic region	modifier	*BRIP1-TBX4*	n.34460131C>A
11	34479592	S	0/0	0/1	0/0	intergenic region	modifier	*BRIP1-TBX4*	n.34479592G>A
11	34488136	S	0/0	0/1	0/0	intergenic region	modifier	*BRIP1-TBX4*	n.34488136A>G
11	34488717	S	0/0	0/1	0/0	intergenic region	modifier	*BRIP1-TBX4*	n.34488717A>G

11	34490833	S	0/0	0/1	0/0	intergenic region	modifier	*BRIP1-TBX4*	n.34490833C>T
11	34493906	S	0/0	0/1	0/0	intergenic region	modifier	*BRIP1-TBX4*	n.34493906A>C
11	34498143	S	0/0	0/1	0/0	intergenic region	modifier	*BRIP1-TBX4*	n.34498143G>A
11	34499474	S	0/0	0/1	0/0	intergenic region	modifier	*BRIP1-TBX4*	n.34499474T>A
11	34502927	S	0/0	0/1	0/0	intergenic region	modifier	*BRIP1-TBX4*	n.34502927C>T
11	34504338	S	0/0	0/1	0/0	intergenic region	modifier	*BRIP1-TBX4*	n.34504338A>G
11	34507424	S	0/0	0/1	0/0	intergenic region	modifier	*BRIP1-TBX4*	n.34507424A>C
11	34508044	S	0/0	0/1	0/0	intergenic region	modifier	*BRIP1-TBX4*	n.34508044C>T
11	34527607	S	0/0	0/1	0/0	intergenic region	modifier	*BRIP1-TBX4*	n.34527607G>A
11	34529068	S	0/0	0/1	0/0	intergenic region	modifier	*BRIP1-TBX4*	n.34529068T>C
11	34538227	S	0/0	0/1	0/0	intergenic region	modifier	*BRIP1-TBX4*	n.34538227G>C
11	34541829	S	0/0	0/1	0/0	intergenic region	modifier	*BRIP1-TBX4*	n.34541829C>T
11	34542068	S	0/0	0/1	0/0	intergenic region	modifier	*BRIP1-TBX4*	n.34542068G>A
11	34544287	S	0/0	0/1	0/0	intergenic region	modifier	*BRIP1-TBX4*	n.34544287A>G
11	34544440	S	0/0	0/1	0/0	intergenic region	modifier	*BRIP1-TBX4*	n.34544440G>A
11	34544768	S	0/0	0/1	0/0	intergenic region	modifier	*BRIP1-TBX4*	n.34544768G>A
11	34546914	I	0/0	0/1	0/0	intergenic region	modifier	*BRIP1-TBX4*	n.34546915delA

11	34550488	S	0/0	0/1	0/0	intergenic region	modifier	*BRIP1-TBX4*	n.34550488C>T
11	34553236	S	0/0	0/1	0/0	intergenic region	modifier	*BRIP1-TBX4*	n.34553236A>G
11	34554088	S	0/0	0/1	0/0	intergenic region	modifier	*BRIP1-TBX4*	n.34554088C>T
11	34554437	S	0/0	0/1	0/0	intergenic region	modifier	*BRIP1-TBX4*	n.34554437C>G
11	34554729	S	0/0	0/1	0/0	intergenic region	modifier	*BRIP1-TBX4*	n.34554729C>A
11	34555749	I	0/0	0/1	0/0	intergenic region	modifier	*BRIP1-TBX4*	n.34555750delA
11	34555964	S	0/0	0/1	0/0	intergenic region	modifier	*BRIP1-TBX4*	n.34555964G>A
11	34555975	S	0/0	0/1	0/0	intergenic region	modifier	*BRIP1-TBX4*	n.34555975G>C
11	34556279	S	0/0	0/1	0/0	intergenic region	modifier	*BRIP1-TBX4*	n.34556279C>T
11	34556790	S	0/0	0/1	0/0	intergenic region	modifier	*BRIP1-TBX4*	n.34556790A>G
11	34561852	S	0/0	0/1	0/0	intergenic region	modifier	*BRIP1-TBX4*	n.34561852G>A
11	34561954	S	0/0	0/1	0/0	intergenic region	modifier	*BRIP1-TBX4*	n.34561954T>C
11	34564286	S	0/0	0/1	0/0	intergenic region	modifier	*BRIP1-TBX4*	n.34564286A>C
11	34564300	S	0/0	0/1	0/0	intergenic region	modifier	*BRIP1-TBX4*	n.34564300A>G
11	34564387	S	0/0	0/1	0/0	intergenic region	modifier	*BRIP1-TBX4*	n.34564387G>C
11	34565151	S	0/0	0/1	0/0	intergenic region	modifier	*BRIP1-TBX4*	n.34565151G>A
11	34566128	S	0/0	0/1	0/0	intergenic region	modifier	*BRIP1-TBX4*	n.34566128T>G

11	34571035	S	0/0	0/1	0/0	intergenic region	modifier	*BRIP1-TBX4*	n.34571035C>T
11	34575201	S	0/0	0/1	0/0	intergenic region	modifier	*BRIP1-TBX4*	n.34575201C>T
11	34575243	S	0/0	0/1	0/0	intergenic region	modifier	*BRIP1-TBX4*	n.34575243T>G
11	34575921	S	0/0	0/1	0/0	intergenic region	modifier	*BRIP1-TBX4*	n.34575921T>A
11	34576610	S	0/0	0/1	0/0	intergenic region	modifier	*BRIP1-TBX4*	n.34576610G>A
11	34581751	S	0/0	0/1	0/0	intergenic region	modifier	*BRIP1-TBX4*	n.34581751C>T
11	34582337	S	0/0	0/1	0/0	intergenic region	modifier	*BRIP1-TBX4*	n.34582337T>C
11	34582869	S	0/0	0/1	0/0	intergenic region	modifier	*BRIP1-TBX4*	n.34582869G>A
11	34586313	S	0/0	0/1	0/0	intergenic region	modifier	*BRIP1-TBX4*	n.34586313G>A
11	34586863	I	0/0	0/1	0/0	intergenic region	modifier	*BRIP1-TBX4*	n.34586864_34586870delGTATAAT
11	34593247	S	0/0	0/1	0/0	intergenic region	modifier	*BRIP1-TBX4*	n.34593247C>T
11	34596895	S	0/0	0/1	0/0	intergenic region	modifier	*BRIP1-TBX4*	n.34596895A>G
11	34598623	S	0/0	0/1	0/0	intergenic region	modifier	*BRIP1-TBX4*	n.34598623G>A
11	34598910	S	0/0	0/1	0/0	intergenic region	modifier	*BRIP1-TBX4*	n.34598910C>A
11	34602133	S	0/0	0/1	0/0	intergenic region	modifier	*BRIP1-TBX4*	n.34602133T>C
11	34713744	I	0/0	0/1	0/0	upstream gene variant	modifier	*TBX2*	c.-2886_-2885insA

11	34716991	S	0/0	0/1	0/0	intergenic region	modifier	*TBX2-ENSECAG00000003698*	n.34716991A>G
11	34937310	S	0/1	0/1	0/0	intergenic region	modifier	*ENSECAG00000003698-BCAS3*	n.34937310G>T
11	34974843	S	0/1	1/1	0/0	downstream gene variant	modifier	*BCAS3*	c.*537G>T
11	35011963	S	0/1	0/1	0/0	intron variant	modifier	*BCAS3*	c.1680-342T>C
11	35373402	S	0/1	0/0	1/1	intergenic region	modifier	*PPM1D-APPBP2*	n.35373402T>C

Table S14. Primer sequences used for genotyping of candidate SNPs on ECA11. The variant located in *KRT25* was genotyped by the use of Kompetitive Allele Specific PCR (KASP). Restriction fragment length polymorphisms were used for genotyping further five variants. Primer pairs, amplicon size (AS) in base pairs (bp), annealing (AT), restriction enzyme and incubation temperature (IT) are shown.

**Gene**	**Polymorphism**	**Forward primer(s) (5'-3')**	**Reverse primer (5'-3')**	**AS (bp)**	**AT (°C)**	**Restriction enzyme**	**IT (°C)**
ENSECAG00000014468	NC_009154.2:g.21338314G>C	CCCAAGGTTTACGATGAAGAG	TTCCCAATAAGAGGTTTCTGC	464	59	MwoI	60
KRTAP16	NC_009154.2:g.21414219G>A	TGCTCATCAAGTCTCTGTGTG	TTTACAGGAGTCAGCACAAGG	408	58	BccI	37
KRT25	NC_009154.2:g.21891160G>A	CATGCAGAACCTCAACGACCG-FAM<br>CCATGCAGAACCTCAACGACCA-VIC	CACATMCTCCAGGTAGGAGGCAA		61/29 cycles	-	-
TOP2A	NC_009154.2:g.22186465C>T	TGTCAATGGTTTAGGGACTTG	CAAGAAGGTAGTTGAAGGTTGG	455	58	HinfI	37
SP6	NC_009154.2:g.24022045C>T	GAGCCGCGCCGCCCTCGGCCTCCCGCAAGC<br>(Mismatch Primer)	AAGAAGAAGCATTTGCACAAC	376	60	HindIII	37
EPN3	NC_009154.2:g.26187590C>T	ATCCTTGCAGACTGTGATCTC	GTCTGGATGGTGTAGAGGTTC	402	58	FauI	55

Table S15. TaqMan gene expression probes used for validation of expression data. Primer sequences, probe sequence and dyes are shown for *KRT25* and *B2M* used as reference gene.

Gene	Forward primer (5'-3')	Reverse primer (5'-3')	TaqMan probe
KRT25	AACGCTGGAGATTGAACTTCAGT	GTTGCCTTCCGTCTCTGTCA	TAGCCACGAAACACTCC (FAM)
B2M	Ec03468700_m1_F	Ec03468700_m1_R	Ec03468700_m1_V<br>AGTTAAGTGGGATCGAGACCTCTAA (VIC)

Table S16. Animals used for morphologic evaluation. Three hair samples from coat, mane and tail of each horse were morphologically investigated.

Breed/population	Coat	Hypotrichosis	NC_009154.2:g. 21891160G>A (KRT25)	NC_009154.2:g. 24022045C>T (SP6)
American Bashkir Curly Horse	Curly	Complete	A/A	C/C
American Bashkir Curly Horse	Curly	Complete	A/A	C/C
American Bashkir Curly Horse	Curly	Complete	A/A	C/C
American Bashkir Curly Horse	Curly	Incomplete	G/A	C/C
American Bashkir Curly Horse	Curly	Incomplete	G/A	C/C
American Bashkir Curly Horse	Curly	Incomplete	G/A	C/C
American Bashkir Curly Horse	Curly	Incomplete	G/A	C/T
American Bashkir Curly Horse	Curly	Incomplete	G/A	C/T
American Bashkir Curly Horse	Curly	Incomplete	G/A	C/T
American Bashkir Curly Horse	Curly	Not at all	G/G	C/T
American Bashkir Curly Horse	Curly	Not at all	G/G	C/T
American Bashkir Curly Horse	Curly	Not at all	G/G	C/T
American Bashkir Curly Horse	Curly	Not at all	G/G	T/T
American Bashkir Curly Horse	Curly	Not at all	G/G	T/T
American Bashkir Curly Horse	Curly	Not at all	G/G	T/T
American Bashkir Curly Horse	Straight	Not at all	G/G	G/G
American Bashkir Curly Horse	Straight	Not at all	G/G	G/G
American Bashkir Curly Horse	Straight	Not at all	G/G	G/G
Quarter Horse	Straight	Not at all	G/G	G/G
Quarter Horse	Straight	Not at all	G/G	G/G
Quarter Horse	Straight	Not at all	G/G	G/G

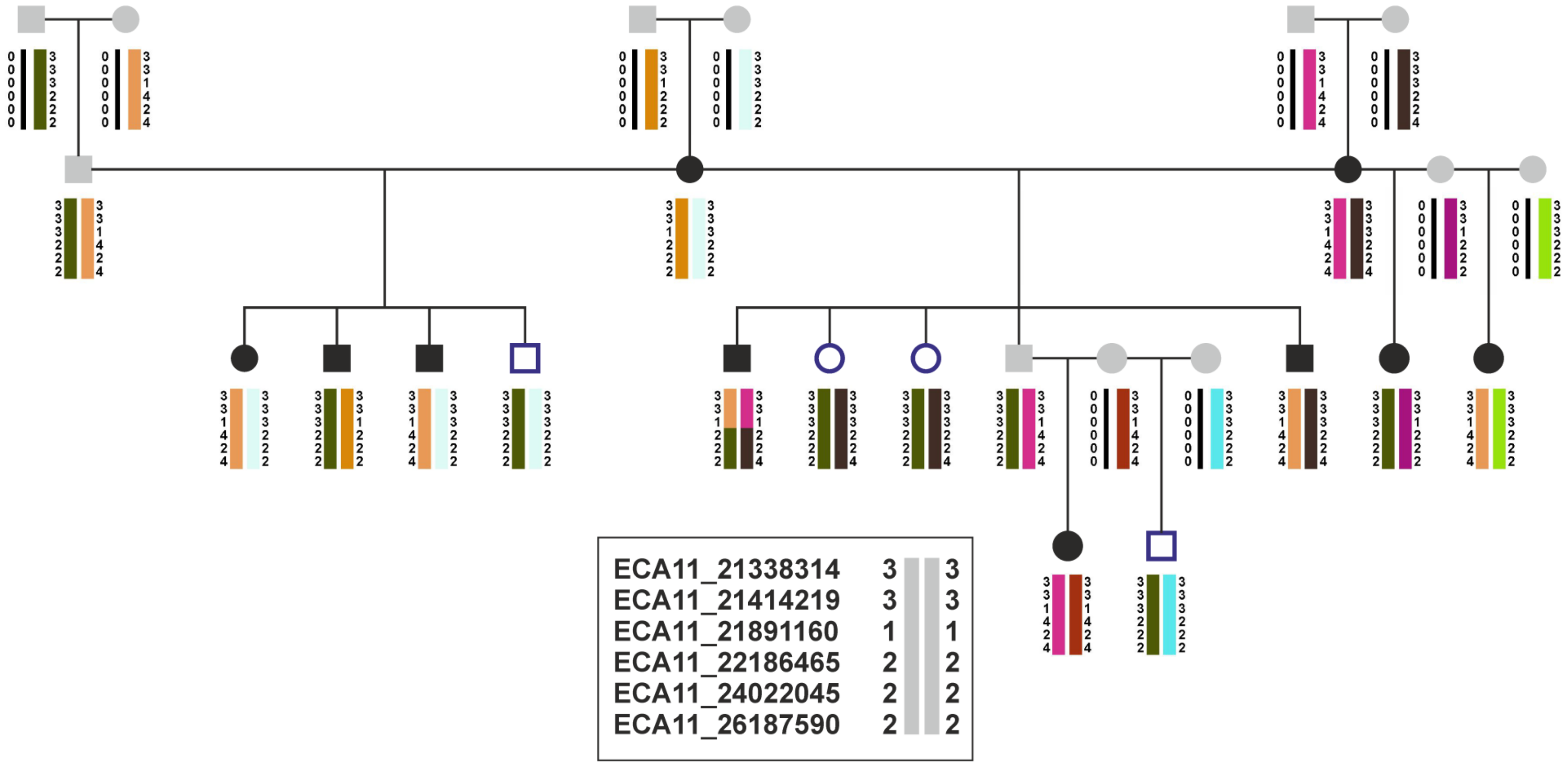

Figure S1. Segregation of haplotypes in ABCH family. Haplotypes based on six missense mutations derived from filtering analysis of whole-genome sequencing data are displayed in an American Bashkir Curly Horse family. Grey symbols represent horses without known phenotype. All curly coated horses (black symbols) harbor one common proximal part of a haplotype block (3-3-1) comprising the first three SNPs whereas straight coated horses (unfilled symbols) show another haplotype block (3-3-3).

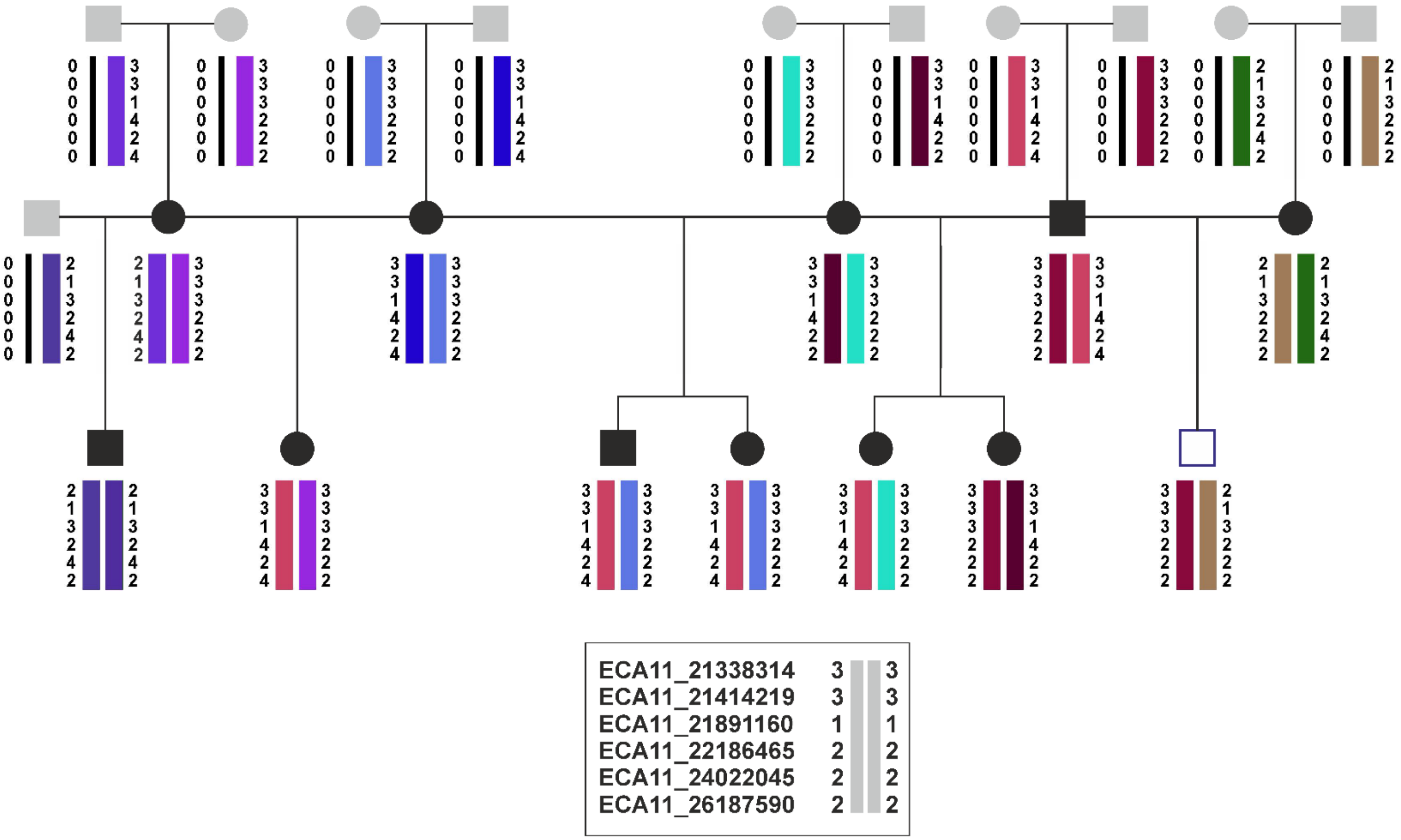

Figure S2. Segregation of haplotypes in ABCH and crossbreed family. Haplotypes based on six missense mutations derived from filtering analysis of whole-genome sequencing data are displayed in a crossbreed family of American Bashkir Curly Horses and Missouri Foxtrotters. Grey symbols represent horses without known phenotype. Three different haplotypes (3-3-1-4-2-4 or 2-1-3-2-4-2 or 3-3-1-4-2-2) can be found that occur only in curly coated horses (black symbols) but not in the straight coated horse (unfilled symbol).

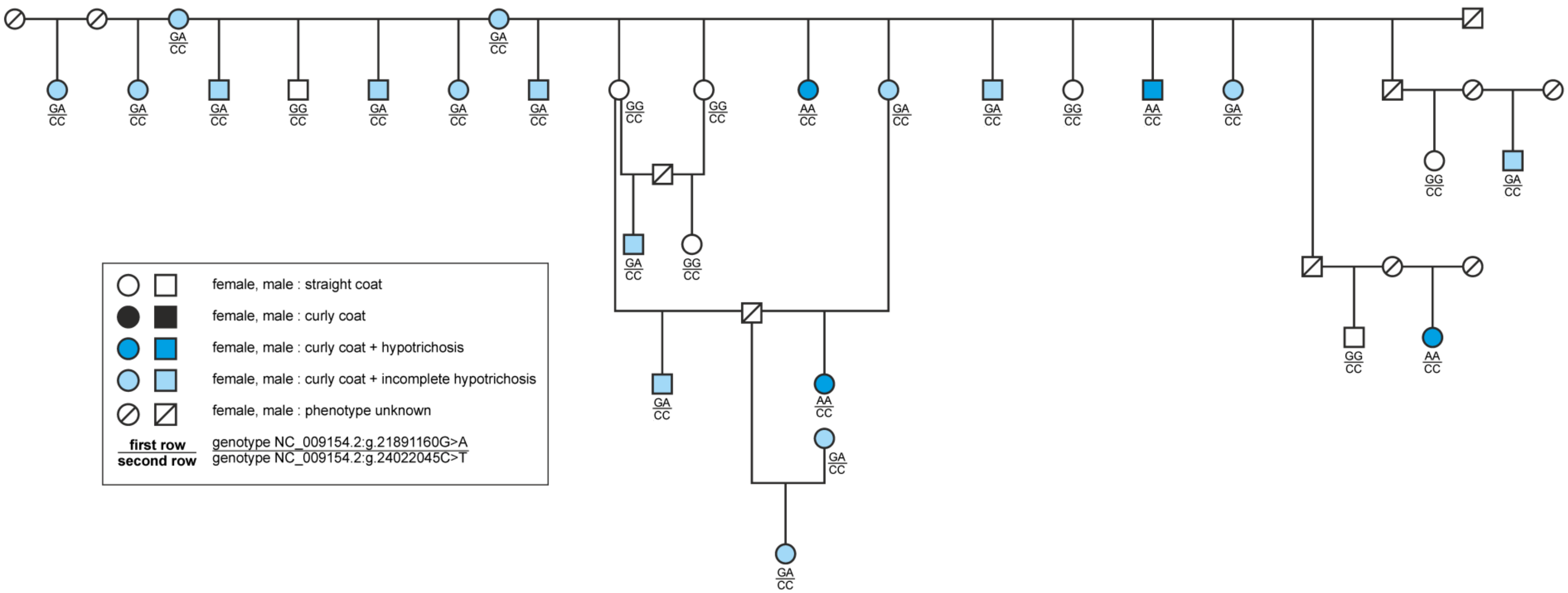

Figure S3. Pedigree of an American Bashkir Curly Horse (ABCH) family I. Circles represent females, squares males. Genotypes of *KRT25* variant (NC_009154.2:g.21891160G>A) and *SP6* variant (NC_009154.2:g.24022045C>T) are assigned. White symbols represent straight coated horses, black symbols curly coated horses without signs of hypotrichosis, blue symbols curly coated horses with complete hypotrichosis and light blue symbols curly coated horses with incomplete hypotrichosis. Individuals that were not phenotyped by us are referred to as unknown.

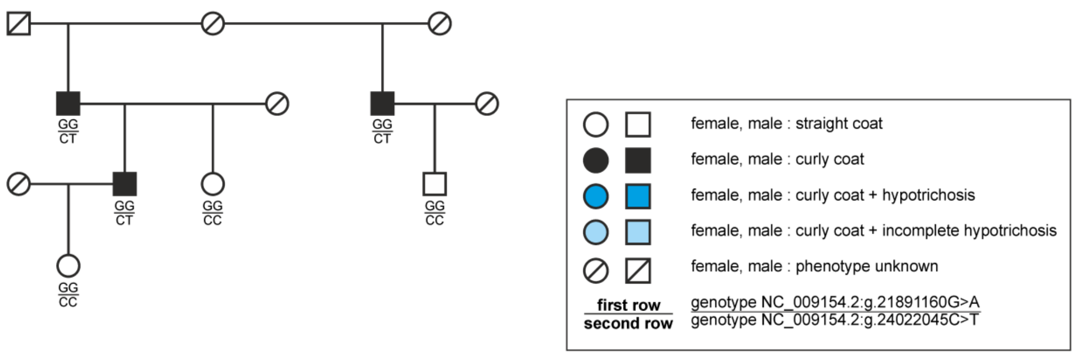

Figure S4. Pedigree of a Missouri Foxtrotter family. Circles represent females, squares males. Genotypes of *KRT25* variant (NC_009154.2:g.21891160G>A) and *SP6* variant (NC_009154.2:g.24022045C>T) are assigned. White symbols represent straight coated horses and black symbols curly coated horses without signs of hypotrichosis. Individuals that were not phenotyped by us are referred to as unknown.

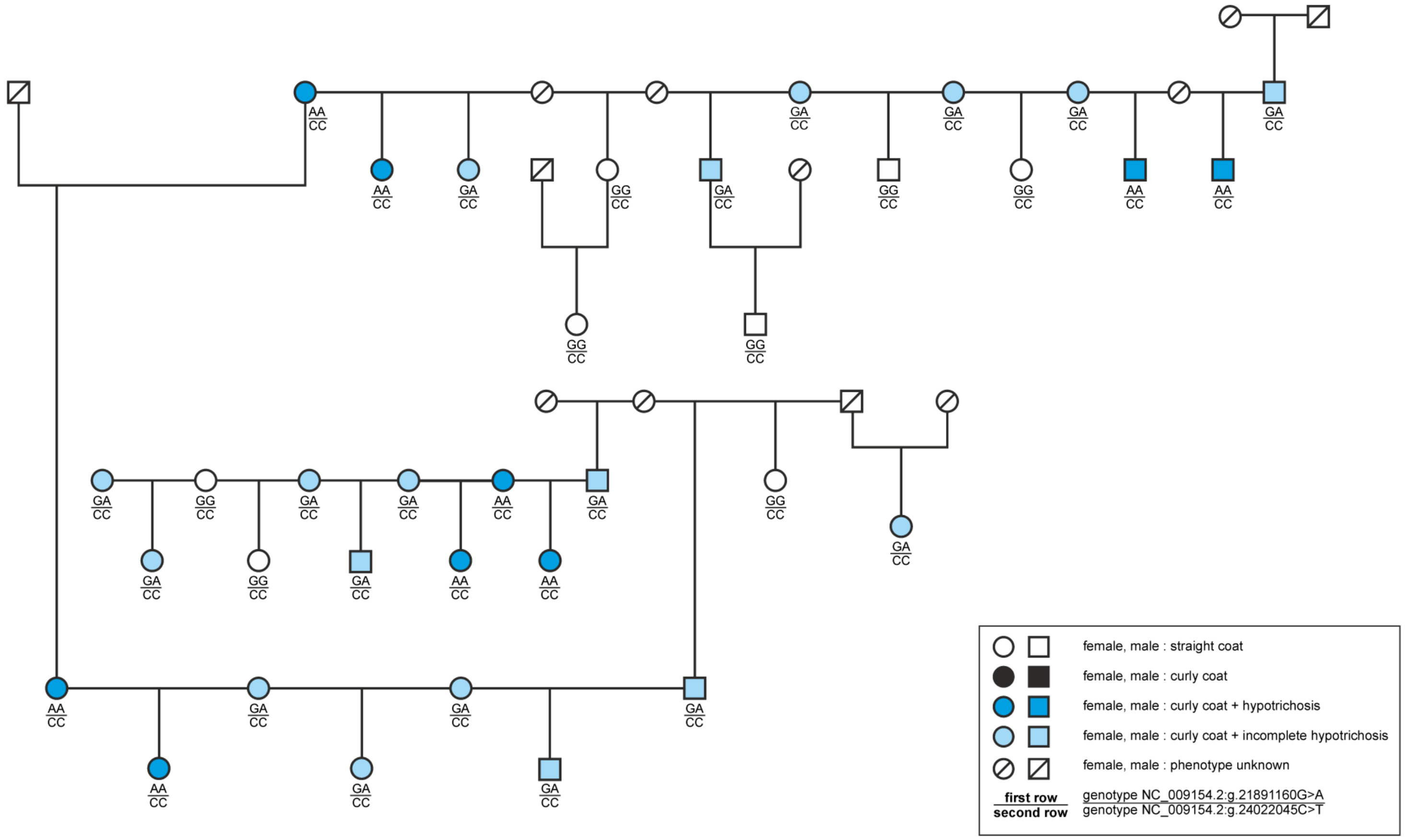

Figure S5. Pedigree of an American Bashkir Curly Horse (ABCH) family II. Circles represent females, squares males. Genotypes of *KRT25* variant (NC_009154.2:g.21891160G>A) and *SP6* variant (NC_009154.2:g.24022045C>T) are assigned. White symbols represent straight coated horses, black symbols curly coated horses without signs of hypotrichosis, blue symbols curly coated horses with complete hypotrichosis and light blue symbols curly coated horses with incomplete hypotrichosis. Individuals that were not phenotyped by us are referred to as unknown.

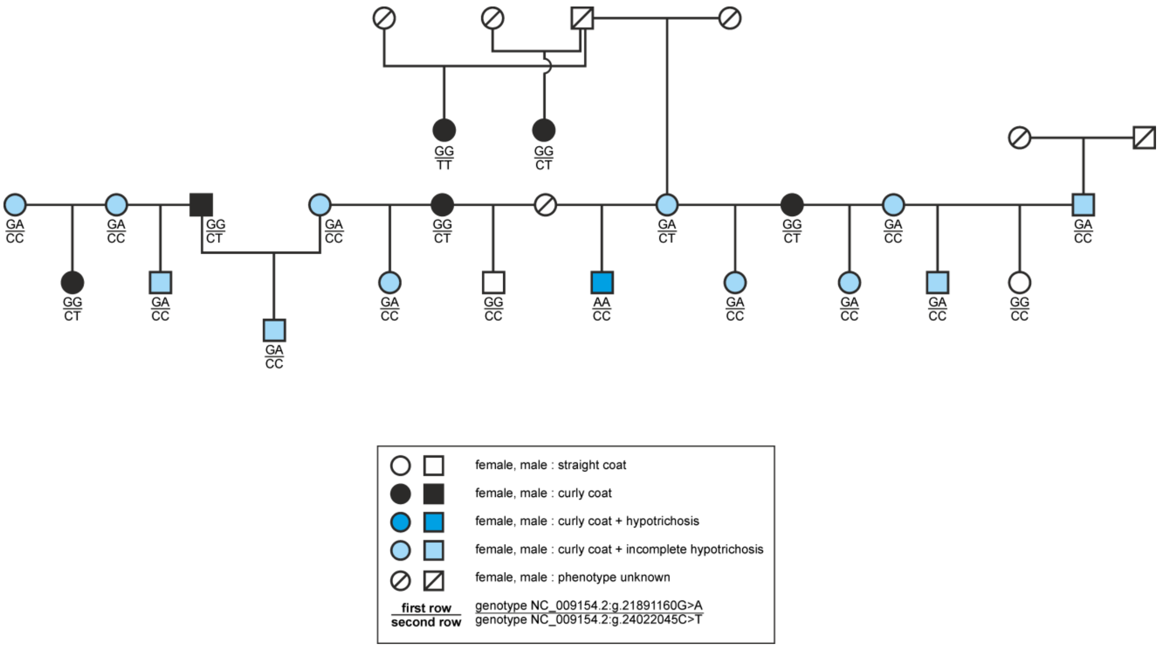

Figure S6. Pedigree of an American Bashkir Curly Horse (ABCH) family III. Circles represent females, squares males. Genotypes of *KRT25* variant (NC_009154.2:g.21891160G>A) and *SP6* variant (NC_009154.2:g.24022045C>T) are assigned. White symbols represent straight coated horses, black symbols curly coated horses without signs of hypotrichosis, blue symbols curly coated horses with complete hypotrichosis and light blue symbols curly coated horses with incomplete hypotrichosis. Individuals that were not phenotyped by us are referred to as unknown.

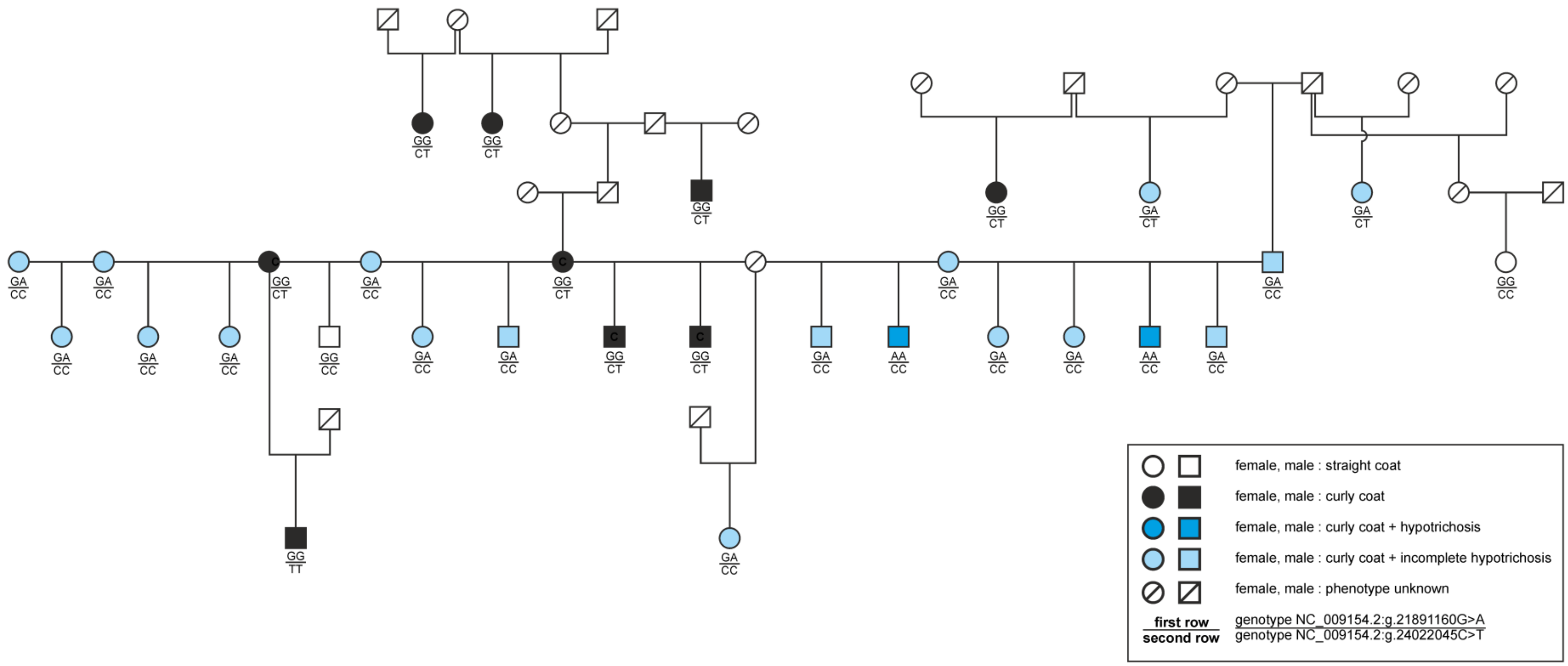

Figure S7. Pedigree of a crossbreed family. Circles represent females, squares males. Genotypes of *KRT25* variant (NC_009154.2:g.21891160G>A) and *SP6* variant (NC_009154.2:g.24022045C>T) are assigned. White symbols represent straight coated horses, black symbols curly coated horses without signs of hypotrichosis, blue symbols curly coated horses with complete hypotrichosis and light blue symbols curly coated horses with incomplete hypotrichosis. Individuals that were not phenotyped by us are referred to as unknown.

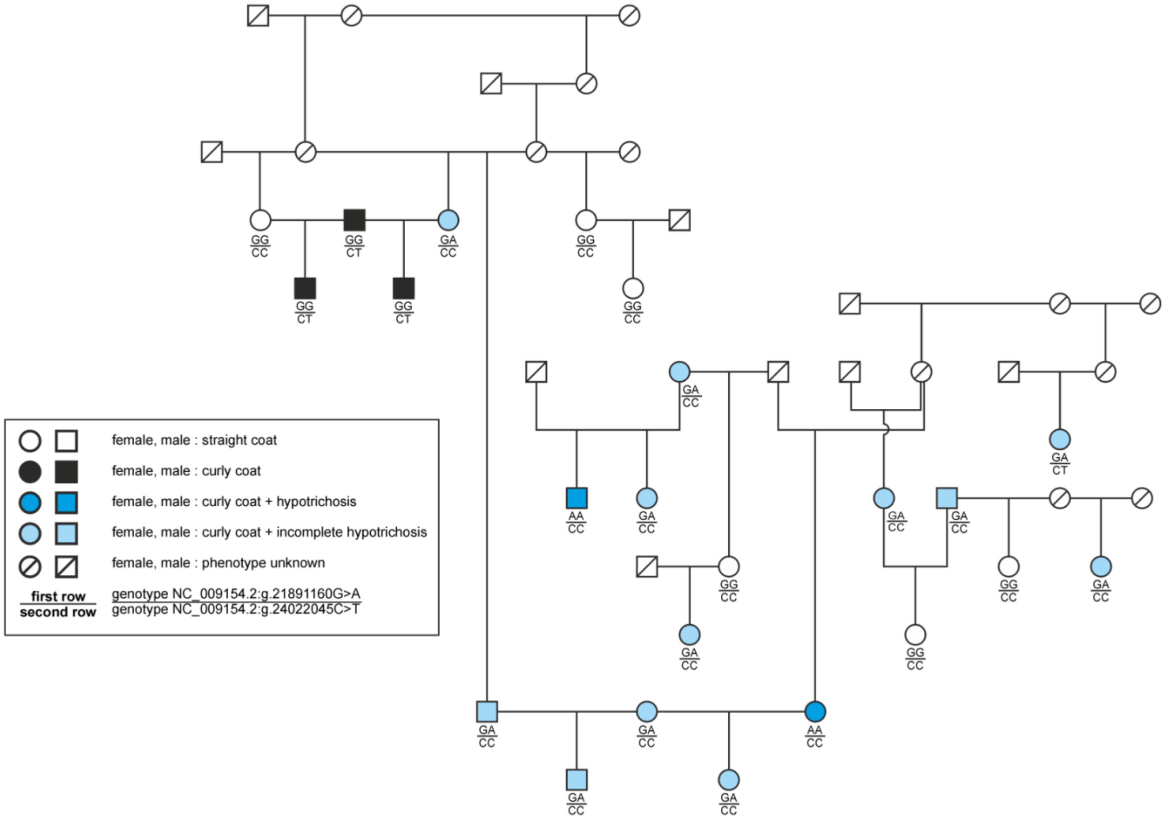

Figure S8. Pedigree of an American Bashkir Curly Horse (ABCH) family IV. Circles represent females, squares males. Genotypes of *KRT25* variant (NC_009154.2:g.21891160G>A) and *SP6* variant (NC_009154.2:g.24022045C>T) are assigned. White symbols represent straight coated horses, black symbols curly coated horses without signs of hypotrichosis, blue symbols curly coated horses with complete hypotrichosis and light blue symbols curly coated horses with incomplete hypotrichosis. Individuals that were not phenotyped by us are referred to as unknown.

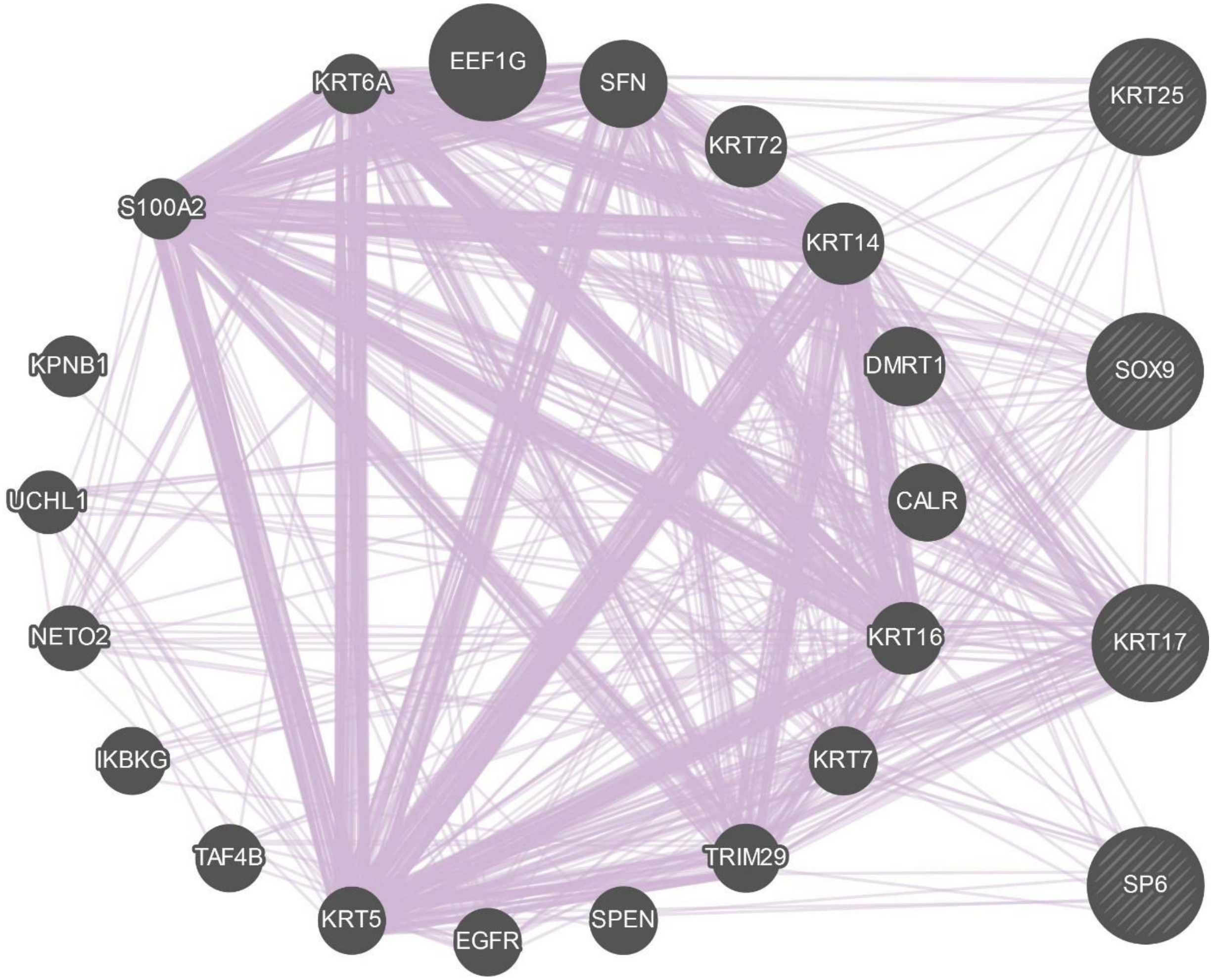

Figure S9. Gene interaction network. Interactions of *KRT17* and *SOX9* showing significant differential expression results in validation analysis of RNA-Seq data with *KRT25* and *SP6*. GeneMANIA interaction network (based on Homo sapiens) shows a co-expression (purple lines) of *SOX9* with *KRT25* and *KRT17*. A dense network of predicted co-expression records is displayed in-between *KRT25*, *SOX9*, *KRT17* and *SP6* with various keratin genes.

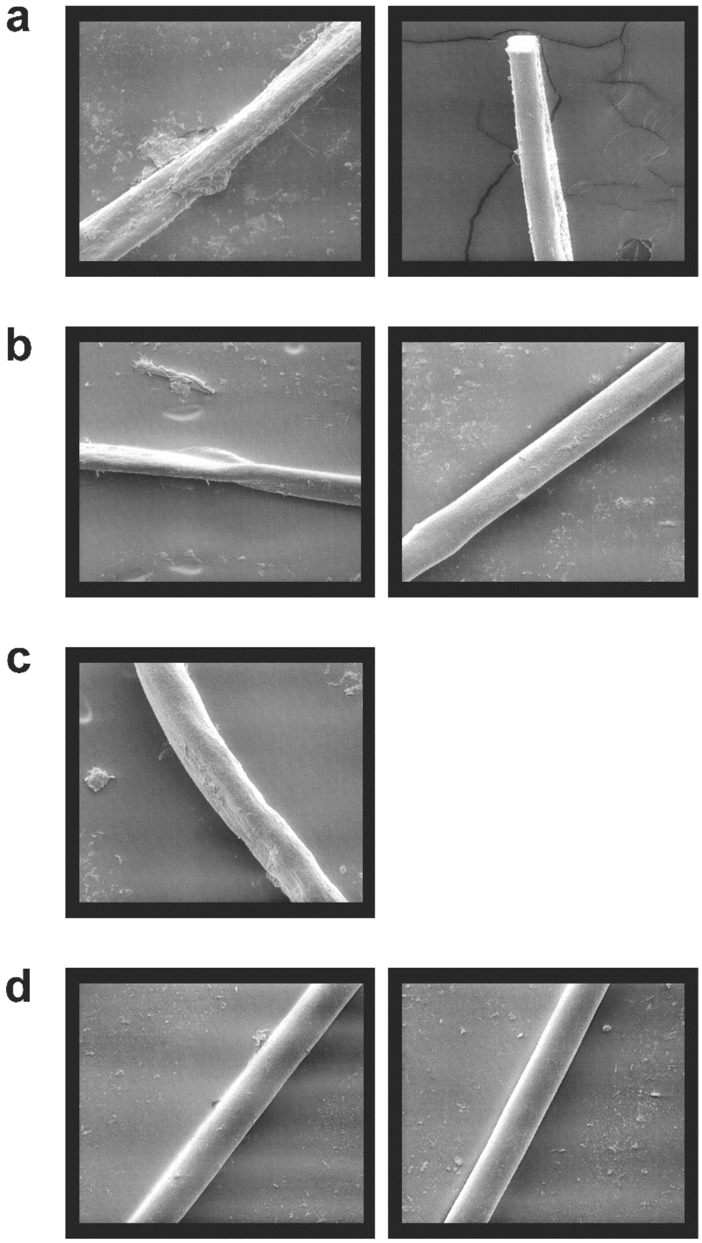

Figure S10. Scanning of curly and straight hair surface. Scanning electron microscopy (SEM) imaging of hair fibers reveals similar characteristics for curly hair in *KRT25* as well as *Sp6* mutant horses (100x, mane). Curly hair mid sections from a horse with a homozygous mutant *KRT25* genotype (a, left) or a heterozygous *KRT25* genotype (a, right) show longitudinal depressions, axial rotation and swellings. Similar findings are displayed in hair samples derived from horses with a homozygous mutant SP6 genotype (b, left) or a heterozygous *SP6* genotype (b, right), as well as a heterozygous *KRT25* and *SP6* genotype (c). Straight hair fibers from an ABCH with a *KRT25* and *SP6* wild type genotype (d, left) and from a QH (d, right) reveal a cylindric shape and no depressions.

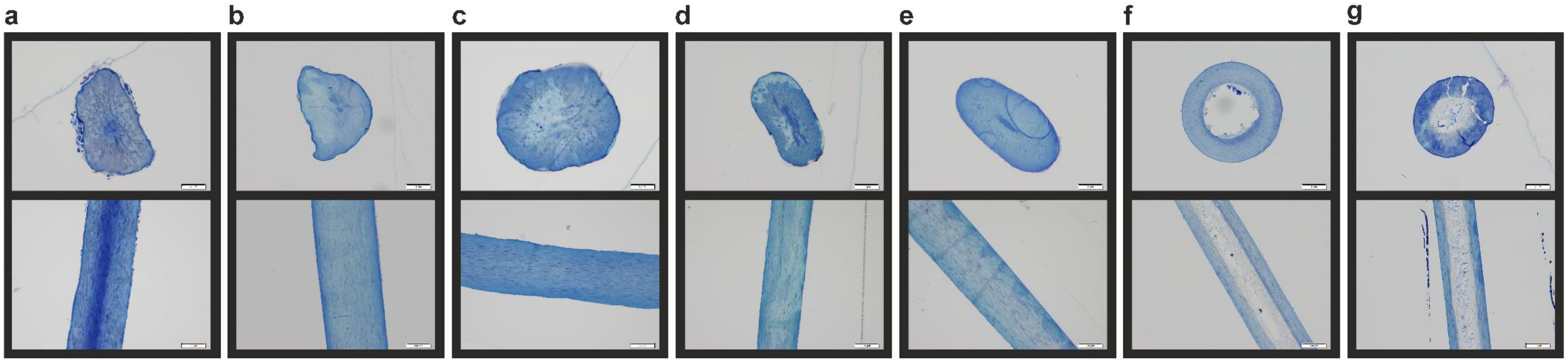

Figure S11. Cross- and longitudinal sections. Light microscopy of cross sections (200x, tail, upper row) and longitudinal sections (100x, tail, lower row) reveals a polymorphic shape without a medulla region in horses homozygous mutant in *KRT25* (a), heterozygous in *KRT25* (b), heterozygous both in *KRT25* and *SP6* (c), homozygous mutant in *SP6* (d), or heterozygous in *SP6* variant (e). Straight hair displays a cylindrical shape with a distinct pronounced medulla in an ABCH with a *KRT25* and *SP6* wild type genotype (f) as well as in a QH (g).
